# Untangling Basal Ganglia Network Dynamics and Function: Role of Dopamine Depletion and Inhibition Investigated in a Spiking Network Model

**DOI:** 10.1523/ENEURO.0156-16.2016

**Published:** 2017-01-12

**Authors:** Mikael Lindahl, Jeanette Hellgren Kotaleski

**Affiliations:** 1 Science for Life Laboratory, School of Computer Science and Communication, KTH Royal Institute of Technology, Box 1031, 17121 Solna, Sweden; 2 Department of Neuroscience, Karolinska Institute, 171 77 Stockholm, Sweden; 3 Stockholm Brain Institute, Karolinska Institute, 171 77 Stockholm, Sweden

**Keywords:** basal ganglia, dopamine, inhibition, network dynamics, Parkinson’s disease, spiking network model

## Abstract

The basal ganglia are a crucial brain system for behavioral selection, and their function is disturbed in Parkinson’s disease (PD), where neurons exhibit inappropriate synchronization and oscillations. We present a spiking neural model of basal ganglia including plausible details on synaptic dynamics, connectivity patterns, neuron behavior, and dopamine effects. Recordings of neuronal activity in the subthalamic nucleus and Type A (TA; arkypallidal) and Type I (TI; prototypical) neurons in globus pallidus externa were used to validate the model. Simulation experiments predict that both local inhibition in striatum and the existence of an indirect pathway are important for basal ganglia to function properly over a large range of cortical drives. The dopamine depletion–induced increase of AMPA efficacy in corticostriatal synapses to medium spiny neurons (MSNs) with dopamine receptor D2 synapses (CTX-MSN D2) and the reduction of MSN lateral connectivity (MSN–MSN) were found to contribute significantly to the enhanced synchrony and oscillations seen in PD. Additionally, reversing the dopamine depletion–induced changes to CTX–MSN D1, CTX–MSN D2, TA–MSN, and MSN–MSN couplings could improve or restore basal ganglia action selection ability. In summary, we found multiple changes of parameters for synaptic efficacy and neural excitability that could improve action selection ability and at the same time reduce oscillations. Identification of such targets could potentially generate ideas for treatments of PD and increase our understanding of the relation between network dynamics and network function.

## Significance Statement

Basal ganglia (BG) are important for selection of behavior, and in Parkinson’s disease (PD), dopamine deficiency causes BG to malfunction. Also, the network dynamic behavior changes, and oscillations and spike synchronization develop. We built a BG network model and used it to better understand how network parameters contribute to function as well as network dynamics, and how functionality can be recovered in the disease state. Our findings improve the general understanding of how BG function and which network parameters are associated with impaired function versus when disease-associated parameter changes can be seen as compensatory. Our results may contribute to novel approaches for the treatment of PD.

## Introduction

Basal ganglia (BG) are critical for the initiation and selection of behaviors and actions, and Parkinson’s disease (PD) caused by dopamine depletion can be linked to inappropriate neural activity in the BG together with impaired function. To improve treatments of PD, we need to understand the underlying neural mechanisms causing BG to malfunction. The effect of dopamine depletion on individual BG network components has been thoroughly studied; however, we still lack an understanding of how the combined effects of altered synaptic efficacy, connectivity, and neural excitability are responsible for the BG dysfunction in PD.

BG have been hypothesized to act as a general action selection device resolving conflicts between potential actions/behaviors competing for restricted resources (Houk and Beiser, 1998; Redgrave et al., 1999; Frank, 2005; Kamali Sarvestani et al., 2011), and in line with this, it has been shown that stimulation of striatum, the main input nucleus of BG, can either promote or inhibit actions (Kravitz et al., 2010; Freeze et al., 2013). Dopamine loss underlying PD (Hornykiewicz, 1966) causes alteration in many places in BG (Cepeda et al., 1993; Shen and Johnson, 2000; Bracci et al., 2002; Hernández-Echeagaray et al., 2004; Hernández et al., 2006; Baufreton and Bevan, 2008; Taverna et al., 2008; Zhou et al., 2009; Humphries et al., 2009a; Chan et al., 2011; Chuhma et al., 2011; Gittis et al., 2011; Miguelez et al., 2012). Computational modeling studies have given us valuable insights into the neural mechanisms behind PD (Terman et al., 2002; Humphries et al., 2006; Kumar et al., 2011; Damodaran et al., 2014, 2015; Corbit et al., 2016), but typically incorporated a subset of the alterations that dopamine depletion causes and investigated the effects in a subset of BG network components. Thus, to get a further understanding of the neural mechanisms in PD, we aimed to study them in a larger network context and try to relate function and dynamic features seen in experiments.

Focal microinjections of the GABA-A antagonist bicuculline in the striatum lead to loss of specificity (LOS) in BG firing patterns (Bronfeld and Bar-Gad, 2011) and cause repetitive motor tics confined to a single or a few muscles (McCairn et al., 2009). Bronfeld and Bar-Gad (2011) showed that LOS actually is a general phenomenon in BG movement disorders, including PD. The activity of the projection neurons (∼95%) in the striatum, the medium spiny neuron (MSN), is controlled by, e.g., recurrent inhibition and feed-forward inhibition from fast spiking neurons (FSNs). Recently, another major source of inhibition from globus pallidus externa (GPe) Type A (TA; arkypallidal) was confirmed (Mallet et al., 2012). The effect of this new pathway on striatal activity has not been studied much in computational models. Thus, to get a better understanding of how the different inhibitory inputs that MSNs receive relate to LOS, there is a need to build a model that accounts for this new pathway.

**Figure 1.**
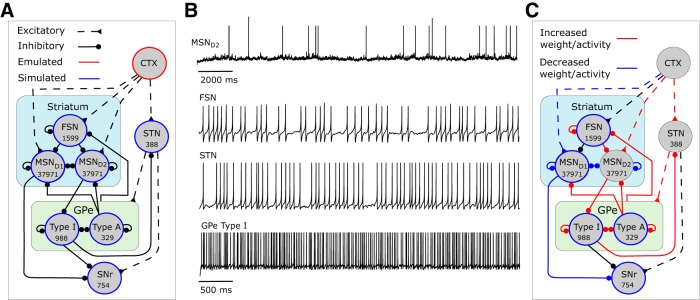
Model description. ***A***, Illustration of the structures included and number of neurons in each simulated nucleus (blue). Solid black lines represent inhibitory synaptic connections; dashed lines represent excitatory synaptic connections. Cortical input was emulated (red). ***B***, Example traces of simulated MSN D2, FSN, STN, and GPe TI neuronal activity. ***C***, Illustration of dopamine-depletion effects on neurons and connections in the network model.

Here we present a quantitative computational model of the BG (Fig. 1). The model includes the striatal network with feedback inhibition from MSNs, feed-forward inhibition from FSNs, and pallidal inhibition from GPe TA neurons, the subthalamic nucleus (STN)–GPe pathway, and the output nucleus substantia nigra reticulata (SNr). Where appropriate, short-term synaptic plasticity is represented. The purpose of building this model was to further test the action selection hypothesis, better understand the underlying neural mechanism of synchrony and oscillations seen in PD, and possibly identify novel targets for treating basal ganglia diseases such as PD. We thus investigate and show how BG network components may support action selection, and how intrastriatal inhibition and alterations in the BG nuclei seen in PD can influence BG network dynamics. Inhibition of MSNs by FSNs and GPe complement the inhibition from MSN collaterals, where the former are most effective during low cortical activity and the latter at higher cortical drive. We predict that the weak but numerous MSN collaterals have a sufficiently strong effect so that active populations of MSNs can significantly suppress the activity of neighboring neurons even when only a small striatal MSN population is bursting after local activation. We find in our model that the strengthening of the drive from cortex to MSN D2, the weakening of the MSN collaterals, and the altered properties of the GPe network, which all are changes associated with dopamine depletion, are very important BG network alterations behind the increased network synchrony and oscillations seen in PD. We see that intrastriatal inhibition can regulate and improve action selection contrast over a range of input strengths, and the activation of STN can delay or prevent action selection in the control network and also improve function in the dopamine-depleted network. Finally, we search for network perturbations of synaptic efficacy and neural excitability that could improve action selection capability or decrease oscillations in PD.

## Materials and Methods

### Network model

The model network consists of a population of MSN D1, MSN D2, FSN, STN, GPe TA, GPe Type I (TI), and SNr neurons modeled as point neurons (Fig. 1A; Mallet et al., 2008). In this study, striatum consisting of MSN D1, MSN D2, and FSNs as well as GPe TA and TI neuron populations (Mallet et al., 2008) were added to a previously published model (Lindahl et al., 2013) in which only the STN, GPe, and SNr network was modeled. Furthermore, we have added known dopamine modulation to neuron types and synapses (Cepeda et al., 1993; Shen and Johnson, 2000; Bracci et al., 2002; Hernández-Echeagaray et al., 2004; Hernández et al., 2006; Baufreton and Bevan, 2008; Taverna et al., 2008; Zhou et al., 2009; Humphries et al., 2009a; Chan et al., 2011; Chuhma et al., 2011; Gittis et al., 2011; Miguelez et al., 2012). All populations received external excitatory uncorrelated Poisson synaptic input to achieve realistic baseline firing rates. Here, cortex would represent the main source of the excitatory input for MSNs, FSNs, and STN, whereas thalamus and brainstem nuclei could be the source of the excitatory input to GPe and SNr (Smith et al., 2010).

A basal ganglia network size of 80,000 neurons was used for all simulations except in simulations in which we study action selection with a network size of 20,000 and when studying inhibition in a striatal network module of ∼3000 neurons. The numbers of neurons used for each nucleus in the model with 80,000 neurons are displayed in Table 1 (see also Fig. 1*A*). The sizes of the nuclei were calculated by using the relative proportion of BG nuclei obtained from the study of absolute numbers of neurons in striatum, STN, GPe, and SNr in rat (Oorschot, 1996) and together with information about the relative distribution of MSN D1, MSN D2 and FSN in striatum [47.5%, 47.5%, and 2%, respectively (Gerfen et al., 2010; Tepper, 2010)] and the relative distribution of GPe TA (arkypallidal) and GPe TI (prototypical) [25% and 75% (Abdi et al., 2015)]. A subpopulation of GPe TI neurons have been shown to project back to FSN (Abdi et al., 2015; Saunders et al., 2016), which we estimated as 10% of the total TI population. With a neural density of 84,900 mm^−3^ (Oorschot, 1996) in striatum, 95% of which are estimated to be MSNs, and radii of the axonal and dendritic arborizations both ∼200 µm (Gerfen et al., 2010), we estimate that there are 2800 MSNs within the volume of the axonal field of one MSN (89,400 × 0.95 × 0.0335). With a volume of 6.5 × 10^−3^ mm (Tepper, 2010) of the axonal arborization of FSNs, we estimate that 540 (89,400 × 0.95 × 0.0065) MSNs are within the axonal field of an FSN. Spatial restrictions were applied only to MSN collateral connectivity and FSN-to-MSN connectivity, based on the data on dendritic and axonal innervation stated above, such that an MSN or FSN could contact only 2800/540 other MSNs assumed to be in closest proximity. All other synaptic connections between and within different nuclei were randomly distributed, since axons and dendritic trees generally project over large target areas in BG (Smith et al., 1998; Sadek et al., 2007; Baufreton et al., 2009; Mallet et al., 2012).

**Table 1.** Network and connection parameters

Name	Value	Description
$N_{network}$	80,000	Network size
$N_{network}^{MSN_{D1}}$	37,971	Size of MSN D1 population
$N_{network}^{MSN_{D2}}$	37,971	Size of MSN D2 population
$N_{network}^{FSN}$	1599	Size of FSN population
$N_{network}^{STN}$	388	Size of STN population
$N_{network}^{GPe_{TA}}$	329	Size of GPe TA population
$N_{network}^{GPe_{TI}}$	988	Size of GPe TI population
$N_{network}^{SNr}$	754	Size of SNr population
$v^{CTX-MSN_{D1}}$	448/546 Hz	Cortical input to MSN D1 (slow-wave/activation)
$v^{CTX-MSN_{D2}}$	592/722 Hz	Cortical input to MSN D2 (slow-wave/activation)
$v^{CTX-FSN}$	646/787 Hz	Cortical input to FSN (slow-wave/activation)
$v^{CTX-STN}$	170/250 Hz	Cortical input to in STN (slow-wave/activation)
$v^{EXT-GPe_{TA}}$	100/200 Hz	External input to GPe TA (slow-wave/activation)
$v^{EXT-GPe_{TI}}$	720/1530 Hz	External input to GPe TI (slow-wave/activation)
$v^{EXT-SNr}$	1800/1800 Hz	External input to SNr (slow-wave/activation)
$a^{CTX-MSN_{D1}}$	0.11	Amplitude oscillations MSN D1 (both states)
$a^{CTX-MSN_{D2}}$	0.11	Amplitude oscillations MSN D2 (both states)
$a^{CTX-FSN}$	0.11	Amplitude oscillations FSN (both states)
$a^{CTX-STN}$	0.11/0.35	Amplitude oscillations STN (slow-wave/activation)
$N_{fan\;in}^{MSN_{D1}-MSN_{D1}}$	364	Number of MSN D1 connections on each MSN D1
$N_{fan\;in}^{MSN_{D1}-MSN_{D2}}$	84	Number of MSN D1 connections on each MSN D2
$N_{fan\;in}^{MSN_{D2}-MSN_{D1}}$	392	Number of MSN D2 connections on each MSN D1
$N_{fan\;in}^{MSN_{D2}-MSN_{D2}}$	504	Number of MSN D2 connections on each MSN D2
$N_{fan\;in}^{FSN-MSN_{D1}}$	16	Number of FSN connections on each MSN neuron
$N_{fan\;in}^{FSN-MSN_{D2}}$	11	Number of FSN connections on each MSN neuron
$N_{fan\;in}^{GPe_{TA}-MSN}$	10	Number of GPe TA connections on each MSN neuron
$N_{fan\;in}^{FSN-FSN}$	10	Number of FSN connections on each FSN neuron
$N_{fan\;in}^{GPe_{TA}-FSN}$	10	Number of GPe TA connections on each FSN neuron
$N_{fan\;in}^{GPe_{TI}-FSN}$	10	Number of GPe TI connections on each FSN neuron
$N_{fan\;in}^{GPe_{TI}-SNr}$	32	Number of GPe connections on each SNr neuron
$N_{fan\;in}^{MSN_{D1}-SNr}$	500	Number of MSN D1 connections on each SNr neuron
$N_{fan\;in}^{STN-SNr}$	30	Number of STN connections on each SNr neuron
$N_{fan\;in}^{MSN_{D2}-GPe_{TI}}$	500	Number of MSN D2 connections on each GPe TI neuron
$N_{fan\;in}^{STN-GPe}$	30	Number of STN connections on each GPe neuron
$N_{fan\;in}^{GPe_{TA}-GPe_{TA}}$	5	Number of GPe TA reciprocal connections
$N_{fan\;in}^{GPe_{TA}-GPe_{TI}}$	5	Number of GPe TA connections on each GPe TI
$N_{fan\;in}^{GPe_{TI}-GPe_{TA}}$	25	Number of GPe TI connections on each GPe TA
$N_{fan\;in}^{GPe_{TI}-GPe_{TI}}$	25	Number of GPe TI reciprocal connections
$N_{fan\;in}^{GPe_{TI}-STN}$	30	Number of GPe TI connections on each STN neuron

To introduce variations into the neural populations, the capacitance (*C*) and spike threshold (*V_T_*/*V_th_*) were assumed to be Gaussian distributed around a mean capacitance, *C*, and spike threshold, *V_T_*/*V_th_*, respectively. As for the standard deviation of the capacitance, we assumed it to be 10% of the mean, and for the spike threshold, the deviation was set to be 1 mV.

### Neuronal firing rates

To ensure realistic population firing rates, we adjusted the Poisson input to each population *v* (Table 1, network and connection parameters). The activity of GPe TA, GPe TI, and STN neurons were matched to Mallet et al. (2008). As for the other populations, it was ensured that their basal firing rates were in the range of values recorded in *in vivo* experiments, e.g., MSN 0.01–2.0 Hz (Miller et al., 2008), FSN 10–20 Hz (Berke et al., 2004; Gage et al., 2010), and SNr 20–35 Hz (Maurice et al., 2003; Gernert et al., 2004; Zahr et al., 2004; Walters et al., 2007).

### Modeling extrinsic inputs to the basal ganglia network

Input to MSNs, FSNs, STN, GPe populations, and SNr were modeled in all simulations as Poisson processes with the frequencies *v* listed in Table 1 for the states denoted, respectively, cortical activation and cortical slow-wave activity. Cortical activation represents the activity following a hindpaw pinch (under urethane anesthesia), whereas slow-wave activity represents the activity at rest (also under urethane anesthesia; Mallet et al., 2008). A 1-Hz frequency modulation (used during both control and dopamine-depletion experiments) was used for generating the cortical slow-wave activity, and a 20-Hz modulation was used to generate beta oscillations (used during dopamine-depletion experiments) during cortical activation. These frequency-modulated inputs were simulated as ± changes in Poisson frequency with a factor *a* (Table 1) such that the instantaneous frequency was changed between v+v∗a and v−v∗a every half cycle of the modulatory input.

For simulations in which we tested action selection capability of the model, the input was modeled as a transient increase in the Poisson inputs to MSNs, FSNs, and STN neurons. The characteristics of MSN activity *in vivo* (in both anesthetized and nonanesthetized preparations) is a low-frequency firing interrupted by bursts (Wilson, 1993). The basal firing rate for MSNs ranged in simulations between 0.01 and 2.0 Hz, whereas spike frequency during the bursts ranged between 17 and 48 Hz (Miller et al., 2008). It has been suggested that dynamic synapses (which are included in the current model) modify action selection outcome differentially at different burst lengths (Lindahl et al., 2013); thus, to reduce the complexity of the interpretation of results, we set the burst length to 100 ms, in line with experiments showing that MSNs usually burst for at least 100 ms (and sometimes up to 1 s; Miller et al., 2008; Gage et al., 2010). This was done in all simulations testing action selection in the model. Action selection simulations consisted of multiple trials, in which each trial consisted of a selection phase with a 100-ms burst in specific MSN populations followed by a 900-ms rest phase with only background input drive to MSNs. For each input combination of two hypothesized competing actions, we ran 80 trials to account for trial variability. To generate a full plot with, e.g., 7 × 7 cortical input combinations (where typically one input was stronger than the other), we had to run 3920 selection trials. For each of the 7 × 7 (49) combinations of competing action pairs, the result was displayed using a pie chart plot (see below). During action selection simulations with an assumed dopamine depletion, we had cortical beta modulation turned on (see above). Threshold passing during an action selection trial was said to have occurred if the mean firing rate of corresponding SNr neurons during the selection phase dropped below 50% of their mean activity in the control model. Thus each selection trial could have four outcomes, and the relative proportion of those were represented in each pie in the pie chart plot: (1) only action 1 was selected; (2) only action 2 was selected; (3) both actions were selected; or (4) neither of the actions was selected.

In simulations in which we tested the effect of striatal inhibition, the input was modeled as a stepwise increase in the amplitude of the simulated cortical input to MSNs, FSNs, and STN.

### Point neuron models used

We used two types of neural models, both hybrid spiking, with one fast and one slow state variable. For FSNs and MSNs, we used the quadratic integrate and fire model with adaptation (Eq. 1; Izhikevich, 2007) and parameters taken from Humphries et al. (2009a,b) (Tables 2 and 4). For STN, GPe, and SNr neurons, we used the adaptive exponential integrate and fire model (Eq 2; Brette and Gerstner, 2005) with parameters taken from Lindahl et al. (2013) (Tables 3, 5 and 6). Both neuron models have a good trade-off between simulation efficacy and ability to capture important dynamic behaviors of neurons (Izhikevich, 2010).

**Table 2.** FSN model parameters (quadratic integrate and fire model)

Name	Value	Description
*a*	0.2 s	Recovery current time constant
*b*	0.025	Voltage dependency of recovery current
*c*	**–**60 mV	Spike reset
*C*	80 pF	Membrane capacitance
*d*	0 pA	Summed recovery current contribution following an action potential
*k*	1	Steady-state voltage dependence
$\beta_{v_{r}}$	**–**0.078	Magnitude of dopamine effect on resting potential *v_t_*
*v_b_*	**–**55 mV	Voltage dependence recovery current
*v_peak_*	25 mV	Spike cutoff
*v_r_*	**–**64.4 mV	Resting potential
*v_th_*	**–**50 mV	Threshold potential

**Table 3.** GPe neuron parameters (adaptive exponential integrate and fire model)

Name	Value	Description
*a*	2.5 nS	Subthreshold adaptation
*b^TI^*	70 pA	Spike-triggered adaptation TI neurons
*b^TA^*	105 pA	Spike-triggered adaptation TA neurons
$\beta_{E_{L}}$	–0.181	Magnitude of D1 effect on resting potential *E_L_*
*C^TI^*	40 pF	Membrane capacitance TI neurons
*C^TA^*	60 pF	Membrane capacitance TA neurons
$\Delta_{T}^{TI}$	1.7 ms	Slope factor of spike upstroke TI neurons
$\Delta_{T}^{TA}$	2.55 ms	Slope factor of spike upstroke TA neurons
*E_L_*	–55.1 mV	Leak reversal potential
*g_L_*	1 nS	Leak conductance
*I_e–TA_*	1 pA	*I_inj_* to obtain *in vitro* firing rate 8 Hz without synaptic input
*I_e–TI_*	12 pA	*I_inj_* to obtain *in vitro* firing rate 18 Hz without synaptic input
τ*_w_*	20 ms	Adaptation time constant
$t^{f}$	15 mV	Spike cut off
*V_r_*	–60 mV	Spike reset
*V_T_*	–54.7 mV	Threshold potential

The following equations control the dynamics of the quadratic integrate-and-fire model with adaptation, where *V* is the membrane potential and *u* is the contribution of the neuron’s slow currents:

$$C\frac{dV}{dt}\;=k\left(V-v_{r}\right)\left(V-v_{\text{th}}\right)-u+I,$$

$$\frac{du}{dt}=a\left(b(V-v_{r})-u\right),$$

(1) $$\text{if }V>v_{peak}\text{ then }v=c\text{ and }u=u+d.$$

Here *C* is the capacitance, *v_r_* and *v*_th_ are the resting and threshold potentials, *I* is a current source, *a* is the recovery current time constant, *b* is the voltage dependence of the recovery current, and *k* is a parameter determining the steady-state current voltage (*I*–*V*) relation. When the membrane potential *V* reaches *v_peak_*, it is reset to *c*, and then the recovery current *u* is updated with *d*.

The following equations control the dynamics of the adaptive exponential integrate-and-fire model, where *V* is the membrane potential and *w* is the contribution of the neuron’s slow currents:

$$C\frac{dV}{dt}=-g_{L}\left(V-E_{L}\right)+g_{L}\Delta_{T}\exp\left(\frac{V-V_{T}}{\Delta_{T}}\right)-w+I,$$

$$\tau_{w}\frac{dw}{dt}=a\left(V-E_{L}\right)-w,$$

(2) $$\text{if }V>t^{f}\text{ then }V=V_{r}\text{ and }w=w+b.$$

Here *C* is the capacitance, *g_L_* is the leak conductance, *E_L_* and *V_T_* are the resting and threshold potentials, Δ*_T_* is the slope factor, *I* is a current source, τ*_w_* is the recovery current time constant, and *a* is the voltage dependence of the recovery current above. When the membrane potential *V* reaches $t^{f}$, it is reset to *V_r_*, and then the recovery current *w* is increased with *b*.

The effect of tonic dopamine level has been incorporated in the model and is regulated by α*_dop_*, a parameter between 0 and 1 representing the relative level of dopamine receptor occupancy, a concept previously used in Humphries et al. (2006, 2009a,b). The dopamine effect on model parameters *p* is modeled by multiplying them with $1+\beta_{par}\phi \left(\alpha_{dop}\right)$ such that $p\leftarrow p\left[1+\beta_{par}\phi \left(\alpha_{dop}\right)\right]$. Here $\beta_{par}\in \left[-5,1.25\right]$ is a scaling coefficient, for corresponding parameter fitted from experiments, that determines the relationship between dopamine receptor occupancy and the magnitude of the effect. In the function $\phi \left(\alpha_{dop}\right)=\alpha_{dop}-\alpha_{0}$, α_0_ = 0.8 is considered to be the normal dopamine level in all the simulations.

### The FSN model

The FSN model shows type 2 dynamics with a nonlinear step from silent to spiking, which is modeled by letting $\dot{u}=a\left[b{\left(V-v_{b}\right)}^{3}-u\right]$ if $v\leq v_{b}$, otherwise $\dot{u}=-au$ (Izhikevich, 2007; Humphries et al., 2009b). This means that the FSN model can exhibit narrow action potentials with rapid, large, and brief afterhyperpolarizations and display irregular firing around spike threshold (if some noise is added; Tepper, 2010). Dopamine has a depolarizing effect on the FSN through D1 type receptor activation. We model this effect as $v_{r}\leftarrow v_{r}\left(1+\beta_{v_{r}}\phi \right)$, where $\beta_{v_{r}}$ was set such that the resting potential at low dopamine ($\alpha_{dop}=0$ ) was 5 mV lower than at high dopamine ($\alpha_{dop}=1$; Bracci et al., 2002; Centonze et al., 2003). The model parameters are listed in Table 2.

### The GPe neuron model

The GPe neuron exhibits membrane oscillations close to spike threshold, causing irregular firing and regular firing at higher depolarizing currents (Nambu and Llinaś, 1994; Cooper and Stanford, 2000) as well as a rebound spiking upon release from hyperpolarization (Nambu and Llinaś, 1994; Cooper and Stanford, 2000). It has a linear current–frequency relationship, with strong spike frequency adaptation at higher input (Cooper and Stanford, 2000; Bugaysen et al., 2010). Dopamine has a depolarizing effect on the GPe neurons by up-regulating the HCN channel responsible for the regular pacemaking of GPe neurons (Chan et al., 2011). We modeled this effect as $E_{L}\leftarrow E_{L}\left(1+\beta_{E_{L}}\phi \right)$, where $\beta_{E_{L}}$ was tuned such that at low dopamine ($\alpha_{dop}=0$), the resting potential was 10 mV lower than at high dopamine ($\alpha_{dop}=1$).

Abdi et al. (2015) showed that TA neurons have a flatter current-frequency curve (Abdi et al. 2015; 60% of the TI slope) compared with TI neurons. To account for this, we set the spike triggered adaptation *b*, spike slope factor Δ*_T_*, and membrane capacitance *C* of TA neurons to 150% of TI neurons (Table 3). For TA and TI neurons to fire at 8 and 18 Hz, respectively, at zero current injection (Abdi et al. 2015), we set $I_{e}^{GPe_{TA}}$ to 1 pA and $I_{e}^{GPe_{TI}}$ to 12 pA. In addition to the input from STN, GPe neurons have been shown to receive input from the central medial and parafascicular nucleus of thalamus (Kincaid et al., 1991; Deschênes, 1996; Yasukawa et al., 2004). We include the assumed contribution from these two structures as a Poisson-type external excitatory background input to TA and TI. The GPe neuron model parameters are listed in Table 3.

### The MSN models

The MSN model captures the prominent long latency to spike discharge of MSNs (Nisenbaum et al., 1994) and that MSN D2 cells have a higher input resistance, due to smaller dendritic surface, and are slightly but significantly more excitable than MSN D1 cells (Kreitzer and Malenka, 2007; Gertler et al., 2008). MSN dopamine receptor D1 activation has a hyperpolarizing effect by increased KIR but also enhances the neural response to depolarizing input, see Gruber et al. (2003). The first effect is modeled as a dopamine-dependent change in the threshold potential as $v_{r}^{MSN_{D1}}\leftarrow v_{r}^{MSN_{D1}}\left(1+\beta_{v_{r}}^{MSN_{D1}}\phi \right)$ and the second as a change in the recovery current as $d^{MSN_{D1}}\leftarrow d^{MSN_{D1}}\left(1+\beta_{d}^{MSN_{D1}}\phi \right)$, and both were set such that the values of *V_r_* and *d* changed with dopamine as in Humphries et al., (2009a). Planert et al. (2013) demonstrate that MSN D2 excitability depends on dopamine concentration; however, interestingly, with low dopamine concentrations (60 µM), argued to be more representative for *in vivo* conditions, no significant (and consistent) change in excitability is seen. Thus we choose to not include any postsynaptic dopamine effect for MSN D2 dopamine receptor activation. The MSN neuron parameters are listed in Table 4.

**Table 4.** MSN D1 and MSN D2 model parameters (quadratic integrate and fire model)

Name	Value	Description
*a*	0.01 s	Recovery current time constant
α	0.032	Magnitude of D2 effect on *k*
*b*	**–**20	Voltage dependency of recovery current
*c*	**–**60 mV	Spike reset
*C*	15.2 pF	Membrane capacitance
$d^{MSN_{D1}}$	66.9 pA	Spike-triggered adaptation MSN D1
$d^{MSN_{D2}}$	91 pA	Spike-triggered adaptation MSN D2
*k*	1	Steady-state voltage dependence
$\beta_{v_{r}}^{MSN_{D1}}$	0.0296	Magnitude of D1 effect on threshold potential *v_t_*
$\beta_{d}^{MSN_{D1}}$	–0.450	Magnitude of D1 effect on recovery current contribution *d*
*v_peak_*	40 mV	Spike cutoff
$v_{r}^{MSN_{D1}}$	**–**78.2 mV	Resting potential MSN D1
$v_{r}^{MSN_{D2}}$	**–**80 mV	Resting potential MSN D2
*v_th_*	**–**29.7 mV	Threshold potential

### The SNr neuron model

The SNr neuron has a linear current frequency relation with spike frequency adaption (Nakanishi et al., 1987; Richards et al., 1997). From holding potential at just below spike threshold, small changes of ∼5 pA in injected current are sufficient to bring the neuron from silent to repetitively firing (Atherton and Bevan, 2005), and the SNr neuron exhibits rebound spiking upon release from hyperpolarization (Nakanishi et al., 1987, 1997). Dopamine has a depolarizing effect on the SNr neurons (Zhou et al., 2009). We modeled this effect as $E_{L}\leftarrow E_{L}\left(1+\beta_{E_{L}}\phi \right)$, where $\beta_{E_{L}}$ was set such that at low dopamine ($\alpha_{dop}=0$), the resting potential was 5 mV lower than at high dopamine ($\alpha_{dop}=1$). The model parameters are listed in Table 5.

**Table 5.** SNr neuron model parameters (adaptive exponential integrate and fire model)

Name	Value	Description
*a*	3 nS	Subthreshold adaptation
*b*	200 pA	Spike-triggered adaptation
$\beta_{E_{L}}$	–0.0896	Magnitude of D1 effect on resting potential *E_L_*
*C*	80 pF	Membrane capacitance
Δ*_T_*	1.8 ms	Slope factor of spike upstroke
*E_L_*	–55.8 mV	Leak reversal potential
*g_L_*	3 nS	Leak conductance
*I_e_*	15 pA	*I_inj_* to obtain *in vitro* firing rate without synaptic input
τ*_w_*	20 ms	Adaptation time constant
$t^{f}$	20 mV	Spike cut off
*V_r_*	–65mV	Spike reset
*V_T_*	–55.2 mV	Threshold potential

### The STN neuron model

The STN neuron can fire at high frequency and has a steep current frequency curve (Bevan and Wilson, 1999; Hallworth et al., 2003). Duration of the afterhyperpolarization after a brief depolarization for ∼500 ms should depend on injected current strength (Bevan and Wilson, 1999). Depolarizing the neuron below –70 mV for a certain period should lead to a rebound burst (Bevan et al., 2000; Hallworth et al., 2003). The hyperpolarization-induced burst of STN neurons was modeled by resetting *V* after a spike to $V_{reset}+\max\left(w-15,20\right)$ with w<0. The parameters of the STN model are listed in Table 6.

**Table 6.** STN neuron parameters (adaptive exponential integrate and fire model)

Name	Value	Description
*a*	0.3 nS	Subthreshold adaptation (below –70) otherwise equal to 0
*b*	0.05 pA	Spike-triggered adaptation
*C*	60 pF	Membrane capacitance
Δ*_T_*	16.2 ms	Slope factor of spike upstroke
*E_L_*	–80.2 mV	Leak reversal potential
*g_L_*	10 nS	Leak conductance
*I_e_*	5 pA	*I_inj_* to obtain *in vitro* firing rate without synaptic input
τ*_w_*	333 ms	Adaptation time constant
$t^{f}$	15 mV	Spike cut off
*V_r_*	–70 mV	Spike reset
*V_T_*	–64.0 mV	Threshold potential

### Synaptic connectivity

Some of the connections (Table 1) in the current model network were obtained from a previously published model (Lindahl et al., 2013). Below we describe the added connections.

For the synapse types MSN D1–MSN D1, MSN D1–MSN D2, MSN D2–MSN D1, and MSN D2–MSN D2, the postsynaptic neurons were estimated to receive from neighboring neurons 364, 84, 393, and 504 synapses, respectively. This is motivated in the following way: within the dendritic and axonal tree of an MSN D1 or MSN D2, there are ∼2800 MSNs (see Network model). The connection probability of MSN D1–MSN D1, MSN D1–MSN D2, MSN D2–MSN D1, and MSN D2–MSN D2 is 13%, 3%, 14%, and 18%, respectively (Taverna et al., 2008); thus, the respective in-degree adds up to 364, 84, 393, and 504 (2800 × 0.13, 0.03, 0.14, or 0.18). Similarly, the number of synapses that MSN D1 and MSN D2 neurons receive from FSNs was estimated as 16 and 11, respectively. We have 60 FSNs within the dendritic tree of an MSN assuming 95% and 2%, respectively, of MSNs and FSNs in striatum (Tepper, 2010; 2800 ÷ 0.95 × 0.02). Then with probability of connection at 27% and 18% for FSN-MSN D1 and FSN-MSN D2 (Gittis et al., 2010; 60 × 0.27 or 0.18), we estimate the number of synapses MSN D1 and MSN D2 neurons receive from FSNs as 16 and 11. FSNs also make synapses onto other FSNs, with 74% probability of contacting a neighboring FSN (Gittis et al., 2010). Because there are 540 MSNs within the axonal field of an FSN, we can estimate the number of FSNs within the axonal field to 12 (560 ÷ 0.95 × 0.02). Thus we estimate that one FSN makes contact with nine other FSNs (0.74 × 12). The MSNs and FSNs were ensured to compile with spatial aspects of biology in which each MSN and FSN only were considered connecting neighboring neurons up to the numbers that each axonal tree contains (2800 and 540, respectively).

Kita and Kitai (1994) and Bevan et al. (1998) found by labeling neurons and tracing axons that a population of GPe neurons (∼25%) project more or less exclusively to striatum. In Mallet et al. (2008) it was shown, based on the discharge pattern during slow-wave sleep, that there exist two types of GPe neurons: TA, which preferentially discharge during the “active” component of the slow 1-Hz cortical oscillation, and TI, which preferentially discharged during the negative “inactive” component. The study suggested that TA preferentially is driven by excitation from STN and that TI is inhibited from the striatum. Chuhma et al. (2011) supported the claim that there is a population of GPe neurons not receiving much input from MSNs by showing that 28% of the GPe neurons in their study were not activated by light stimulation of striatal fibers. In recent studies (Mallet et al., 2012, 2016; Gittis et al., 2014; Abdi et al., 2015) it was shown that TA and TI have different structural connectivity in which TA strongly connects to striatum (but not to STN or to basal ganglia output nuclei) and TI preferably connects to BG output nuclei (and STN). Abdi et al. (2015) also showed that a subpopulation of TI cells (estimated to 10%) indeed projects to striatum. We thus include in our model TA neurons that project to striatum, but not to STN. TI neurons are also included which, in contrast to TA project to STN, receive input from striatum, and where one out of every tenth TI neuron projects back to striatum. Specifically, in the striatum, TA neurons project to both MSNs and FSNs, whereas TI neurons project only to FSNs in accordance with the literature (Glajch et al., 2016; Saunders et al., 2016).

Mallet et al. (2012) established that a TA neuron gives rise to ∼10,000 synaptic boutons in striatum, but the relative innervations of FSNs and MSNs were not determined. There are up to 100 times more MSNs than FSNs in striatum, and therefore we assume that for each connection GPe makes with FSNs, it contacts 100 MSNs. Furthermore, we assumed that 1000 is the maximal number of MSNs that a single TA neuron connects to. Then, on average, each MSN receives input from 10 GPe (1000 connections ÷ 100 = MSN/TA ratio), and thus each TA cell is assumed to make 10 boutons on each MSN. We then have that 1 TA cell makes 10,000 boutons onto MSNs (10 × 1000 = 10,000 synaptic boutons). For an FSN, we assume that it also receives input from 10 TA and that each TA makes 10 boutons on each FSN. Then 1 TA cell makes 100 boutons on FSNs (10 × 10 = 100). We then have that in total 1 TA cell makes 10,100 boutons in striatum (which is reasonable). TA makes a dense and specific innervation over a large area in striatum (Mallet et al., 2012); therefore, we did not put any spatial restriction on which striatal cells TA could make contact with. For TI to FSN, we likewise assumed that each FSN received input from 10 TI cells.

Both TA and TI neurons receive input from STN (Kita and Kitai, 1994), and TI gives rise to four times more collaterals than TA (Mallet et al., 2012). There are three TI neurons for each TA neuron in GPe (75% TI versus 25% TA in GPe). So for each TI connection, we have 12 (4 × 3) as many TI connections onto an average GPe cell compared with a TA connection. It is reasonable to then assume that the majority of synapses onto specifically the TA cells come from TI neurons, since TI synapses are a magnitude more numerous than TA. However, it is still possible that TA neurons receive more connections from neighboring TA if there is some sort of connection preference rule at hand. Our base estimate in this study was that of 30 incoming GPe collaterals, 5 will be from TA and 25 from TI. Later in this article, we challenge this assumption to see the effect on network dynamics with different fan-in from TI and TA to TA.

### Synapse models

To model static synapses, we used a standard conductance-based exponential decay model equation and the Tsodyks–Markram model (Tsodyks et al., 1998) to capture synaptic short-term plasticity (Hanson and Jaeger, 2002; Sims et al., 2008; Connelly et al., 2010; Gittis et al., 2010; Planert et al., 2010).

For static synapses, Eq. 3 is used when a presynaptic spike arrives. The conductance *g* is updated with *g*_0_ and then, in between the spikes, the conductance decays toward zero with time constant τ*_syn_*. The postsynaptic current is given by $I=g\left(E_{rev}-V\right)$, where *V* is the membrane potential:

(3) $$\frac{dg}{dt}=-\frac{g}{\tau_{syn}}+g_{0}\delta \left(t-t_{spike}\right)$$

The NMDA-dependent magnesium block was captured by multiplying the synaptic current with *B* (Eq. 4; Humphries et al., 2009b):

(4) $$B\left(v\right)=\frac{1}{1+\frac{{\left[{\text{Mg}}^{2+}\right]}_{2}}{3.57}\exp\left(-v0.062\right)}$$

To model a frequency-dependent synapse (Table 8), the Tsodyks model (Tsodyks et al., 1998) was used (Eqs. 5 and 6) with the common FD formalism (Abbott et al., 1997; Dittman et al., 2000; Abbott and Regehr, 2004; Puccini et al., 2007). The FD formalism dictates that the synaptic strength is updated by the product of facilitating (F) and depressing (D) variables/factors. This description shows quantitatively good approximations of experimentally measured synapse dynamics (Tsodyks and Markram, 1997; Markram et al., 1998; Planert et al., 2010; Klaus et al., 2011; Lindahl et al., 2013). The model formalism assumes a finite pool of synaptic resources in active (*y*), inactive (*z*), and recovered (*x*) states. At rest, *y* and *z* are 0 and *x* is 1. Depression occurs because some of the resources remain for a while in the inactive state before entering the recovered state, with a rate determined by the recovery time constant τ*_rec_*. The facilitation is modeled by *u*, which is a variable that is stepwise increased at each spike with the product of the utilization factor *U* and 1 – *U* (*U* is between 0 and 1) and decays exponentially toward 0 with time constant τ*_fac_* in between spikes (Eq. 5). The resources in the active state *y* are increased with the product of the variables *x* and *u* (capturing depression and facilitation, respectively) and are then quickly inactivated by decaying toward zero with time constant τ*_syn_* (Eq. 6). The postsynaptic conductance is proportional to the fraction of resources in the active state and is given by $g=g_{0}y$, with the resulting postsynaptic current $I_{syn}=g\left(E_{rev}-V\right)$:

(5) $$\frac{du}{dt}=-\frac{u}{\tau_{fac}}+U\left(1-u\right)\delta \left(t-t_{spike}\right),$$

$$\frac{dx}{dt}=\frac{z}{\tau_{rec}}-ux\delta \left(t-t_{spike}\right),$$

$$\frac{dy}{dt}=-\frac{y}{\tau_{syn}}+ux\delta \left(t-t_{spike}\right),$$

(6) $$\frac{dz}{dt}=\frac{y}{\tau_{syn}}-\frac{z}{\tau_{rec}}.$$

Several of the synaptic parameters (Tables 7 and 8) were obtained from Lindahl et al. (2013). Below we explain how we derived CTX–MSN and the parameters from and to GPe TA and GPe TI. From Ellender et al. (2011), we estimate that the mean conductance evoked when stimulating cortical MSN fibers to 1.1 nS, since the peak of excitatory postsynaptic potential (EPSC) was ∼90 pA at holding potential –80 mV, with AMPA reversal potential at 0 mV (90 pA ÷ 80 mV = 1.1 nS). Assuming that the unitary strength of an AMPA synapse is below measured mean, we set it to 0.5 nS. Moyer et al. (2007) have estimated that the ratio of conductance sizes between AMPA and NMDA is 2:1. The conductance size is estimated here by the time constant multiplied with the peak conductance of a synapse; thus for the MSN AMPA synapse with a synaptic decay constant of 12 ms (Ellender et al., 2011), the conductance size equals 6 (0.5 × 12), and for the MSN D2 NMDA synapse with a synaptic time constant of 160 ms (Moyer et al., 2007; Humphries et al., 2009b), the maximal conductance has to be 0.019, since the conductance size for NMDA should equal 3 (0.019 × 160), to give an AMPA and NMDA conductance size ratio of 2:1. Furthermore, Humphries et al. (2009a) estimated the parameter used for MSN D1 NMDA as ∼6 times that for MSN D2 NMDA under normal dopamine conditions to resemble the behavior seen in more detailed MSN models. Thus we set the MSN D1 NMDA to 0.11 (0.019 × 6). These differences between how MSN D1 and D2 NMDA properties are represented in this phenomenological point neuron model should not be interpreted literally; rather, it captures the dopamine-dependent modulation of the direct and indirect pathways.

**Table 7.** Basic synaptic model parameters

Name	Value	Source
$\tau_{ampa}^{CTX-FSN}$	12 ms	n.d., set as for CTX-MSN
$g_{ampa}^{CTX-FSN}$	0.5 nS	n.d., set as for CTX-MSN
$t_{delay}^{CTX-FSN}$	2.5 ms	Jaeger and Kita, 2011
$E_{rev}^{CTX-FSN}$	0 mV	n.d., set as for CTX-MSN
$\tau_{gaba}^{FSN-FSN}$	6 ms	Gittis et al., 2010
$g_{gaba}^{FSN-FSN}$	1 nS	Several times weaker than FSN-MSN (Gittis et al., 2010)
$t_{delay}^{FSN-FSN}$	1.7 ms	n.d., same as FSN-MSN
$E_{rev}^{FSN-FSN}$	–74 mV	n.d., same as MSN-MSN
$\tau_{gaba}^{GPe_{TI}-FSN}$	17 ms	Saunders et al., 2016
$g_{gaba}^{GPe_{TI}-FSN}$	2 nS	n.d., estimated
$\tau_{gaba}^{GPe_{TA}-FSN}$	66 ms	Glajch et al., 2016
$g_{gaba}^{GPe_{TA}-FSN}$	0.51 nS	n.d., estimated
$t_{delay}^{GPe-FSN}$	7.0 ms	n.d., same as MSN-GPe Park et al., 1982
$E_{rev}^{GPe-FSN}$	–74 mV	n.d., same as MSN-MSN
$\tau_{ampa}^{EXT-GPe}$	5 ms	n.d.
$g_{gaba}^{EXT-GPe}$	0.5 nS	n.d.
$t_{delay}^{EXT-GPe}$	5 ms	n.d.
$E_{rev}^{EXT-GPe}$	0 mV	n.d.
$\tau_{gaba}^{GPe-GPe}$	5 ms	Shen et al., 2008
$g_{gaba}^{GPe-GPe_{TI}}$	1.3 nS	Lindahl et al., 2013
$g_{gaba}^{GPe-GPe_{TA}}$	0.33 nS	25% of GPe-GPe TI
$t_{delay}^{GPe-GPe}$	1 ms	n.d.
$E_{rev}^{GPe-GPe}$	–65 mV	n.d., assumed as for MSN D2-GPe
$\tau_{gaba}^{MSN_{D2}-GPe}$	6 ms	Shen et al., 2008
$g_{gaba}^{MSN_{D2}-GPe}$	2 nS	Constrained by Shen et al., 2008
$t_{delay}^{MSN_{D2}-GPe}$	7 ms	Park et al., 1982
$E_{rev}^{MSN_{D2}-GPe}$	–65 mV	Rav-Acha et al., 2005
$\tau_{ampa}^{STN-GPe_{TI}}$	12 ms	Hanson and Jaeger, 2002
$g_{ampa}^{STN-GPe_{TI}}$	0.35 nS	Lindahl et al., 2013
$g_{ampa}^{STN-GPe_{TA}}$	0.11 nS	30% of STN-GPe TI
$t_{delay}^{STN-GPe_{TI}}$	2 ms	Jaeger and Kita, 2011
$E_{rev}^{STN-GPe_{TI}}$	0 mV	n.d.
$\tau_{ampa}^{CTX-MSN}$	12 ms	Ellender et al., 2011
$g_{ampa}^{CTX-MSN_{D1}}$	0.5 nS	Humphries et al., 2009a,b; Ellender et al., 2011
$g_{ampa}^{CTX-MSN_{D2}}$	0.5 nS	Humphries et al., 2009a,b; Ellender et al., 2011
$\tau_{nmda}^{CTX-MSN}$	160 ms	Humphries et al., 2009a,b; Ellender et al., 2011
$g_{nmda}^{CTX-MSN_{D1}}$	0.11 nS	Humphries et al., 2009a,b; Ellender et al., 2011
$g_{nmda}^{CTX-MSN_{D2}}$	0.019 nS	Humphries et al., 2009a,b; Ellender et al., 2011
$t_{delay}^{CTX-MSN}$	2.5 ms	Jaeger and Kita, 2011
$E_{rev}^{CTX-MSN}$	0 mV	Humphries et al., 2009a
$\tau_{gaba}^{MSN-MSN}$	8 ms	Gittis et al., 2010
$g_{gaba}^{MSN_{D1}-MSN_{D1}}$	0.15 nS	Constrained by Koo^s^ et al., 2004; Taverna et al., 2004, 2008
$g_{gaba}^{MSN_{D1}-MSN_{D2}}$	0.375 nS	Constrained by Koos et al., 2004; Taverna et al., 2004, 2008
$g_{gaba}^{MSN_{D2}-MSN_{D1}}$	0.45 nS	Constrained by Koos et al., 2004; Taverna et al., 2004, 2008
$g_{gaba}^{MSN_{D2}-MSN_{D2}}$	0.35 nS	Constrained by Koos et al., 2004; Taverna et al., 2004, 2008
$t_{delay}^{MSN-MSN}$	1.7 ms	Taverna et al., 2004
$E_{rev}^{MSN-MSN}$	–74 mV	Koo^s^ et al., 2004
$\tau_{gaba}^{FSN-MSN}$	11 ms	Koos et al., 2004
$g_{gaba}^{FSN-MSN}$	6 nS	Taverna et al., 2004
$t_{delay}^{FSN-MSN}$	1.7 ms	n.d., assumed as for MSN
$E_{rev}^{FSN-MSN}$	–74 mV	Koós and Tepper, 1999
$\tau_{gaba}^{GPe_{TA}-MSN_{D1}}$	87 ms	Glajch et al., 2016
$\tau_{gaba}^{GPe_{TA}-MSN_{D2}}$	76 ms	Glajch et al., 2016
$g_{gaba}^{GPe-MSN_{D1}}$	0.04 nS	n.d., estimated
$g_{gaba}^{GPe-MSN_{D2}}$	0.08 nS	Twice as TA-MSN D1 (Glajch et al., 2016)
$t_{delay}^{GPe-MSN}$	7.0 ms	n.d., set as for MSN-GPe (Park et al., 1982)
$E_{gaba}^{GPe-MSN}$	–74 mV	n.d., set as for MSN and FSN synapses
$\tau_{ampa}^{EXT-SNr}$	5 ms	n.d.
$g_{ampa}^{EXT-SNr}$	0.5 nS	n.d.
$t_{delay}^{EXT-SNr}$	5 ms	n.d.
$E_{rev}^{EXT-SNr}$	0 mV	n.d.
$\tau_{gaba}^{MSN_{D1}-SNr}$	5.2 ms	Connelly et al., 2010
$g_{gaba}^{MSN_{D1}-SNr}$	2 nS	Constrained by Connelly et al., 2010
$t_{delay}^{MSN_{D1}-SNr}$	7 ms	Connelly et al., 2010
$E_{rev}^{MSN_{D1}-SNr}$	–80 mV	Connelly et al., 2010
$\tau_{gaba}^{GPe-SNr}$	2.1 ms	Connelly et al., 2010
$g_{gaba}^{GPe-SNr}$	76 nS	Connelly et al., 2010
$t_{delay}^{GPe-SNr}$	3 ms	Nakanishi et al., 1991
$E_{rev}^{GPe-SNr}$	–72 mV	Connelly et al., 2010
$\tau_{ampa}^{STN-SNr}$	12 ms	n.d., assume as for STN-GPe (Hanson and Jaeger, 2002)
$g_{ampa}^{STN-SNr}$	0.91 nS	Lindahl et al., 2013
$t_{delay}^{STN-SNr}$	4.5 ms	Shen and Johnson, 2006; Ammari et al., 2010
$E_{rev}^{STN-SNr}$	0 mV	n.d.
$\tau_{ampa}^{CTX-STN}$	4 ms	Baufreton et al., 2005
$g_{ampa}^{CTX-STN}$	0.25 nS	n.d.
$\tau_{nmda}^{CTX-STN}$	160 ms	Same as for MSN
$g_{nmda}^{CTX-STN}$	0.00625 nS	Same AMPA-NMDA ratio as for MSN
$t_{delay}^{CTX-STN}$	2.5 ms	Fujimoto and Kita, 1993; Jaeger and Kita, 2011
$E_{rev}^{CTX-STN}$	0 mV	n.d.
$\tau_{gaba}^{GPe-STN}$	8 ms	Baufreton et al., 2005
$g_{gaba}^{GPe_{TI}-STN}$	0.08 nS	Lindahl et al., 2013
$t_{delay}^{GPe-STN}$	1 ms	Jaeger and Kita, 2011
$E_{rev}^{GPe-STN}$	–84 mV	Baufreton et al., 2009

**Table 8.** Parameters for facilitating and depressing Tsodyks synapse models

Synapse	*U*	τ*_rec_*	τ*_fac_*	Source
*FSN − MSN*	0.29	902 ms	53 ms	Planert et al., 2010
*FSN − FSN*	0.29	902 ms	53 ms	Gittis et al., 2010; Planert et al., 2010
*GPe − SNr*	0.196	969 ms	0 ms	Lindahl et al., 2013
*MSN_D1_ − SNr*	0.0192	623 ms	559 ms	Lindahl et al., 2013
*MSN_D2_ − SNr*	0.24	11 ms	73 ms	Lindahl et al., 2013
*STN − SNr*	0.35	800 ms	0 ms	Lindahl et al., 2013
*GPe_TA_ − FSN*	0.29	902 ms	53 ms	n.d., assumed same as *FSN − MSN*
*GPe_TI_ − FSN*	0.29	902 ms	53 ms	n.d., assumed same as *FSN − MSN*

Experiments suggest that both TA and TI neurons connect to FSNs, whereas only TA neurons connect to MSNs (Mallet et al. 2012; Smith et al., 1998; Glajch et al. 2016; Saunders et al. 2016). Specifically, Glajch et al. (2016) showed, by light stimulation of GPe axons going to striatum, that the inhibitory postsynaptic currents (IPSCs) in FSNs were considerable larger than the IPSCs in MSN D1 and MSN D2 (600 vs. 60 and 134 pA), and Saunders et al. (2016) measured IPSCs in FSNs by light stimulation of axons from TI neurons and observed large IPSCs in FSNs (437 pA). Here we assume that TA neurons connect twice as strongly to MSN D2 than to MSN D1 and that TA and TI neurons connect strongly to FSNs (Table 7) in accordance with Glajch et al. (2016) and Saunders et al (2016). Additionally, we assume that the TA and TI synapses onto FSN are depressing (Corbit et al., 2016; Glajch et al., 2016). Because we did not have an estimate of the parameters for the depression, we used the same parameters as for FSN to MSN (Table 8). We also point out that the synapse dynamics of TA and TI neurons in striatum seem to differ. Saunders et al. (2016) recorded significantly faster synapse dynamics for TI cells onto FSNs compared with those Glajch et al. (2016) measured for the synaptic response in FSN, MSN D1, and MSN D2 when stimulating GPe axons (17 vs. 66, 87, and 76 ms). The slice experiments in Glajch et al. (2016) were conducted at room temperature (∼21°C), whereas Saunders et al. (2016) conducted their experiments at higher temperature (∼31°C). It is commonly assumed that increased temperature can speed up such processes. Thus, the recording temperature could account for some of these differences. Here we assume that the synapse dynamics between TA and TI cells indeed differ and that TI synapses in striatum have faster dynamics than synapses from TA cells.

In Lindahl et al. (2013), the conductance between GPe–GPe, GPe–STN, and STN–GPe neurons were respectively estimated to be 1.3, 0.35, and 0.08 nS. In this study, the GPe TI neurons are assumed to correspond to the GPe neurons in Lindahl et al. (2013) since they have similar projection patterns. Thus GPe–GPe TI, GPe TI–STN, and STN–GPe TI were set to these values. TA neurons do not receive any inhibitory input from MSN D2. To maintain realistic firing rates in TA, we lowered STN–TA to 30% of STN–TI. However, to properly explain changes in TI and TA rates for slow-wave and activation, we also had to lower the weights of GPe–GPe TA to 25% of GPe–GPe TI.

Numerous connections in BG are modified by dopamine (Cepeda et al., 1993; Shen and Johnson, 2000; Bracci et al., 2002; Hernández-Echeagaray et al., 2004; Hernández et al., 2006; Baufreton and Bevan, 2008; Taverna et al., 2008; Chuhma et al., 2011; Gittis et al., 2011; Miguelez et al., 2012). The dopamine parameters related to the synaptic connectivity are listed in Table 9. Below we describe how we adapted the model to this.

**Table 9.** Synaptic dopamine parameters

Name	Value	Source
$\beta_{I_{GABA}}^{FSN-FSN}$	–1.27	Humphries et al., 2009a
$\beta_{I_{GABA}}^{GPe-FSN}$	–0.53	Glajch et al., 2016
$\beta_{I_{GABA}}^{GPe-GPe}$	–0.83	Miguelez et al., 2012
$\beta_{I_{GABA}}^{MSN_{D2}-GPe_{TI}}$	–0.83	Chuhma et al., 2011
$\beta_{I_{AMPA}}^{STN-GPe}$	–0.45	Hernández et al., 2006
$\beta_{I_{NMDA}}^{CTX-MSN_{D1}}$	1.04	Humphries et al., 2009b
$\beta_{I_{AMPA}}^{CTX-MSN_{D2}}$	–0.26	Humphries et al., 2009b
$\beta_{N_{fan\;in}}^{FSN-MSN_{D2}}$	–0.90	Gittis et al., 2011
$\beta_{I_{GABA}}^{MSN-MSN}$	0.88	Taverna et al., 2008
$\beta_{N_{fan\;in}}^{MSN-MSN}$	0.88	Taverna et al., 2008
$\beta_{I_{GABA}}^{GPe_{TA}-MSN_{D1}}$	–1.22	Glajch et al., 2016
$\beta_{I_{GABA}}^{GPe_{TA}-MSN_{D1}}$	–1.15	Glajch et al., 2016
$\beta_{I_{GABA}}^{MSN_{D1}-SNr}$	0.56	Chuhma et al., 2011
$\beta_{I_{AMPA}}^{CTX-STN}$	–0.45	Shen and Johnson, 2000
$\beta_{I_{GABA}}^{GPe-STN}$	–0.24	Baufreton and Bevan, 2008

### Dopamine effects on synapses onto FSNs

Dopamine has a weakening effect on GABA synapses (Bracci et al., 2002), by activation of D2 receptors. We modeled this by multiplying *I_GABA_* for FSN–FSN and GPe–FSN with, respectively, $1+\beta_{I_{GABA}}^{FSN-FSN}\phi$ and $1+\beta_{I_{GABA}}^{GPe-FSN}\phi$, where $\beta_{I_{GABA}}^{FSN-FSN}$ and $\beta_{I_{GABA}}^{GPe-FSN}$ were set such that the IPSC amplitude at low dopamine ($\alpha_{dop}=0$) were 2.7 (Humphries et al., 2009b) and 1.6 (Glajch et al., 2016) times the IPSC amplitude at high dopamine ($\alpha_{dop}=1$).

### Dopamine effects on synapses onto GPe neurons

Dopamine depletion–dependent weakening of GPe–GPe synapses has been shown experimentally (Miguelez et al., 2012). This does not seem to involve dopamine receptor effects; instead, it is hypothesized that the change is due to maladaptive homeostasis. We modeled this by multiplying *I_GABA_* for GPe–GPe with $1+\beta_{I_{GABA}}^{GPe-GPe}\phi$, where $\beta_{I_{GABA}}^{GPe-GPe}$ was set such that the IPSC amplitude at low dopamine ($\alpha_{dop}=0$) was two times the IPSC amplitude at high dopamine ($\alpha_{dop}=1$) estimated from Miguelez et al. (2012).

It has been shown that dopamine leads to a decrease in synaptic efficacy of MSN–GPe synapses through D2 activation in rats (Ingham et al., 1997; Cooper and Stanford, 2001; Chuhma et al., 2011). We modeled this by multiplying *I_GABA_* for MSN D2–GPe TI with $1+\beta_{I_{GABA}}^{MSN_{D2}-GPe_{T1}}\phi$, where $\beta_{I_{GABA}}^{MSN_{D2}-GPe_{T1}}$ was set such that the IPSC amplitude at low dopamine ($\alpha_{dop}=0$) was two times the IPSC amplitude at high dopamine ($\alpha_{dop}=1$) estimated from Chuhma et al. (2011).

Dopamine administration reduces STN–GPe EPSCs (Hernández et al., 2006). We modeled this by multiplying *I_AMPA_* for all STN–GPe synapses with $1+\beta_{I_{AMPA}}^{STN-GPe}\phi$, where $\beta_{I_{AMPA}}^{STN-GPe}$ was set such that the EPSC amplitude at low dopamine ($\alpha_{dop}=0$) was 1.5 times the EPSC amplitude at high dopamine ($\alpha_{dop}=1$) as estimated from Hernández et al., (2006).

### Dopamine effects on synapses onto MSNs

Dopamine has a strengthening effect on CTX–MSN D1 NMDA currents, whereas it has a weakening effect on CTX–MSN D2 AMPA currents (Cepeda et al., 1993; Levine et al., 1996; Hernández-Echeagaray et al., 2004). We modeled this by multiplying *I_NMDA_* for CTX–MSN D1 by $1+\beta_{I_{NMDA}}^{CTX-MSN_{D1}}\phi$ and multiplying *I_AMPA_* for CTX–MSN D2 by $1+\beta_{I_{AMPA}}^{CTX-MSN_{D2}}\phi$. $\beta_{I_{NMDA}}^{CTX-MSN_{D1}}$ was set such that the EPSC amplitude at low dopamine ($\alpha_{dop}=0$) was 0.14 times the EPSC amplitude at high dopamine ($\alpha_{dop}=1$), and $\beta_{I_{AMPA}}^{CTX-MSN_{D2}}$ was set such that the EPSC amplitude at low dopamine ($\alpha_{dop}=0$) was 1.27 times the EPSC amplitude at high dopamine ($\alpha_{dop}=1$); both parameter values were obtained from Humphries et al. (2009b).

Dopamine depletion leads to an increase in connections between FSN–MSN D2 but not FSN–MSN D1 (Gittis et al., 2011). We modeled this by multiplying $N_{FSN-MSN_{D2}}$ by $1+\beta_{N_{FSN-MSN_{D2}}}\phi$ such that the number of connections at low dopamine ($\alpha_{dop}=0$) was two times the number of connection at basal dopamine ($\alpha_{dop}=0.8$) as estimated from Gittis et al. (2011).

Dopamine depletion leads to a dramatic decrease in the connectivity between MSNs (Taverna et al., 2008). We modeled this by multiplying *I_GABA_* for MSN–MSN by $1+\beta_{I_{GABA}}^{MSN-MSN}\phi$ and multiplying $N_{MSN-MSN}$ for MSN–MSN by $1+\beta_{N_{MSN-MSN}}\phi$. $\beta_{I_{GABA}}^{MSN-MSN}$ was set such the IPSC at low dopamine ($\alpha_{dop}=0$) was 0.25 times the size of the IPSC at high dopamine ($\alpha_{dop}=1$), and $\beta_{N_{MSN-MSN}}$ was set such that the number of MSN collaterals at low dopamine ($\alpha_{dop}=0$) were 0.25 times the number MSN collaterals at high dopamine ($\alpha_{dop}=1$); both parameters were estimated from Taverna et al. (2008).

Dopamine has an weakening effect on TA–MSN synapses (Glajch et al., 2016). We modeled this by multiplying *I_GABA_* for TA–MSN D1 and TI–MSN D2 with, respectively, $1+\beta_{I_{GABA}}^{GPe_{TA}-MSN_{D1}}\phi$ and $1+\beta_{I_{GABA}}^{GPe_{TA}-MSN_{D2}}\phi$, where $\beta_{I_{GABA}}^{GPe_{TA}-MSN_{D1}}$ and $\beta_{I_{GABA}}^{GPe_{TA}-MSN_{D2}}$ were set such that the IPSC amplitude at low dopamine ($\alpha_{dop}=0$) was 2.6 and 2.5 times (Glajch et al., 2016) the IPSC amplitude at high dopamine ($\alpha_{dop}=1$).

### Dopamine effects on synapses onto SNr neurons

Dopamine D1 receptor activation facilitates MSN–SNr synapses (Chuhma et al., 2011). We modeled this by multiplying *I_GABA_* with $1-\beta_{I_{GABA}}^{MSN_{D1}-SNr}\alpha_{dop}$, where $\beta_{I_{GABA}}^{MSN_{D1}-SNr}$ was set such that the IPSC amplitude at low dopamine ($\alpha_{dop}=0$) was 0.5 times the amplitude at high dopamine ($\alpha_{dop}=1$) estimated from Chuhma et al. (2011).

Dopamine receptor D1 activation has a facilitating effect, whereas dopamine receptor D2 activation has a depressing effect on STN–SNr EPSC (Ibañez-Sandoval et al., 2006). Thus it is not clear whether dopamine enhances or weakens STN–SNr synapses. Here we assumed that dopamine activation did not change the amplitude of STN–SNr EPSC.

### Dopamine effects on synapses onto STN neurons

Weakening of CTX–STN synapses by dopamine (Shen and Johnson, 2000) was modeled by multiplying *I_AMPA_* and *I_NMDA_* with $1-\beta_{I_{AMPA}}^{CTX-STN}\phi$, where $\beta_{I_{AMPA}}^{CTX-STN}$ was set such that EPSC amplitude at low dopamine ($\alpha_{dop}=0$) was 2.5 times the amplitude at high dopamine ($\alpha_{dop}=1$) estimated from Kreiss et al. (1997) and Magill et al. (2001). With this value of $\beta_{I_{AMPA}}^{CTX-STN}$, the firing rate of STN neurons in the network increased ∼100% when removing dopamine, which is in agreement with experiments (Magill et al., 2001; Mallet et al., 2008).

The results by Baufreton and Bevan (2008) suggest that dopamine causes a small but significant decrease in GPe–STN synaptic efficacy at 10- to 50-Hz firing rate. This was modeled by multiplying the IPSC by $1-\beta_{I_{GABA}}^{GPe-STN}\phi$. $\beta_{I_{GABA}}^{GPe-STN}$ was set such that the synaptic conductance without dopamine ($\alpha_{dop}=0$) was 1.25 times the synaptic conductance at maximal dopamine level ($\alpha_{dop}=1$) estimated from Baufreton and Bevan (2008).

### Data analysis

Spike trains were sampled at 256 Hz as in Mallet et al. (2008) when computing coherence. The Hanning window was set to 128 and 2048 ms such that we got frequency resolution for cortical beta and slow-wave activity at 1 and 0.125 Hz as in Mallet et al. (2008). For significance levels for coherence, we used a method from Halliday et al. (1995).

To calculate the phase relation between two neurons, we first smoothed the raw spike trains with a bandpass filter at 0.5–1.5 Hz and 15–25 Hz, respectively, for slow-wave and beta activity. Then we applied the Hilbert transform on the smoothed data to obtain the instantaneous phases, and finally got the phase relation between two neurons by subtracting their instantaneous phases from each other. The result was plotted in a histogram with 100 bins between –π and π.

Firing rate is calculated from time bins equaling 1000/256 (∼4 ms; assuming the same sampling frequency as in Mallet et al, 2008).

To quantify synchrony in BG neurons, we used the Fano factor as a measure (Kumar et al., 2008, 2011). The Fano factor (Eq. 7) of a population *FF*(*pop*) is defined as the variance of the total population firing rate *V*(*pop*) divided by mean of the population firing rate *E*(*pop*). The sampling frequency in Mallet et al., (2008) was 256 Hz; to match this, we used a bin size of 1000/256 ms (∼4 ms) to calculate population firing rates. Poisson processes have a Fano factor of 1. A Fano factor >1 then means that some neurons must fire in a more synchronized manner, i.e., there are more spike events from different neurons occurring close in time than a Poisson process would predict:

(7) FF(pop)=V[pop]/E[pop].

To estimate the strength of beta oscillations in a population, we used the fact that oscillations introduce peaks in the power spectrum density (Kumar et al., 2011). Power spectrum, *S*, was calculated from the average over the spectra of individual neuron spike trains. The oscillation index used to capture this, *OI*, was defined as relative power in a frequency band:

(8) $$OI\left[pop\right]=\frac{\int_{a}^{b}S{\left(f\right)}_{pop}df}{\int_{0}^{Fs/2}S{\left(f\right)}_{pop}df},$$

where *a* and *b* were set to respectively 15 and 25 Hz, a typical frequency band for beta oscillation. The sample frequency *Fs* was 256 Hz as in Mallet et al. (2008).

### Implementation

The simulations were run using the NEST simulator (Gewaltig and Diesmann, 2007; RRID:SCR_002963). Simulations ran on a CRAY XC30 system. The simulation of a network with 80,000 neurons for 10 s took 30 min on 80 cores. The network was built using PyNest, which is a Python interface to the NEST simulator. The model is available for download at github (https://github.com/mickelindahl/bgmodel).

## Results

### Constraining and validating the systems-level BG model with statistics on GPe and STN firing rates, coefficients of variation, coherences, and phase relations

A massive body of work in this study has been to review and compile model data from a large collection of experimental papers and integrate the information into a BG network model. First and foremost, we were concerned with constraining the model to experimental data on connectivity and nuclei sizes (Table 1), synaptic properties (Tables 7 and 8), neuron properties (Tables 2–6), and effects of dopamine depletion (Table 9 and Fig. 1*C*). Two models were used in this study, one with an intact dopamine system (control model) and one representing dopamine-depleted rats after oxidopamine (6-OHDA) treatment (lesioned model). In the current study, one goal was to validate the model against the statistics in Mallet et al. (2008) by comparing model data and experimental data regarding firing rates, coefficients of variation (CVs) of interspike intervals, coherence, and phase shift for and between STN and GPe TI and TA neurons.

**Figure 2.**
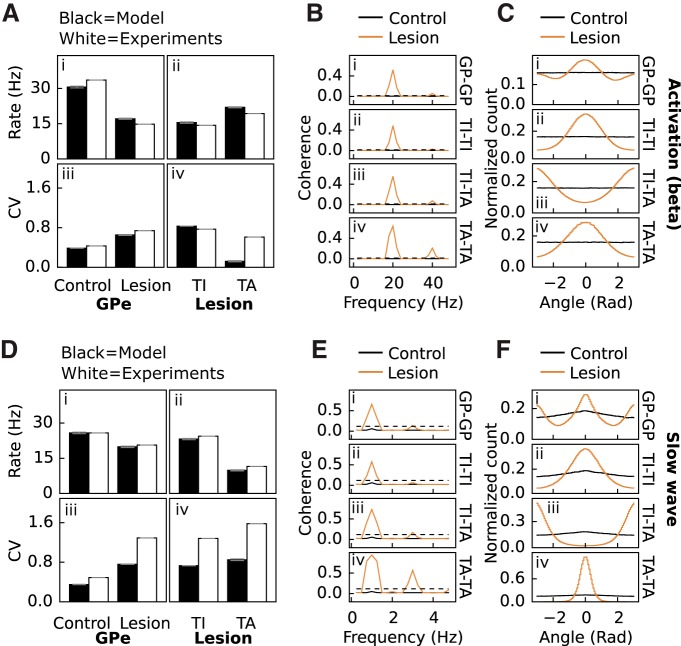
Model validation using GPe as a readout. ***A***, Firing rate and CV of GPe neurons with cortical beta activity used as input for the model, and comparisons with experiments (from Mallet et al, 2008). ***i***, ***iii***, Firing rate and CV of randomly selected GPe neurons in the control and lesioned (dopamine set to 0) network. ***ii***, ***iv***, Firing rate and CV for GPe TI and GPe TA neurons in the lesioned network. ***B***, Coherence of GPe neurons in the control and lesioned network when activated with cortical beta input. ***i–iv***, Coherence for random GPe versus GPe, specifically GPe TI versus GPe TI, GPe TI versus GPe TA, and GPe TA versus GPe TA. Black dotted line shows significance of *p* = 0.05. ***C***, Phase relationship of GPe neurons in the control and lesioned network when activated with cortical beta input. ***i–iv***, Phase relationship for random GPe versus GPe, GPe TI versus GPe TI, GPe TI versus GPe TA, and GPe TA versus GPe TA. ***D***, ***E***, and ***F***, same as ***A***, ***B***, and ***C***, but for cortical slow-wave input.

**Figure 3.**
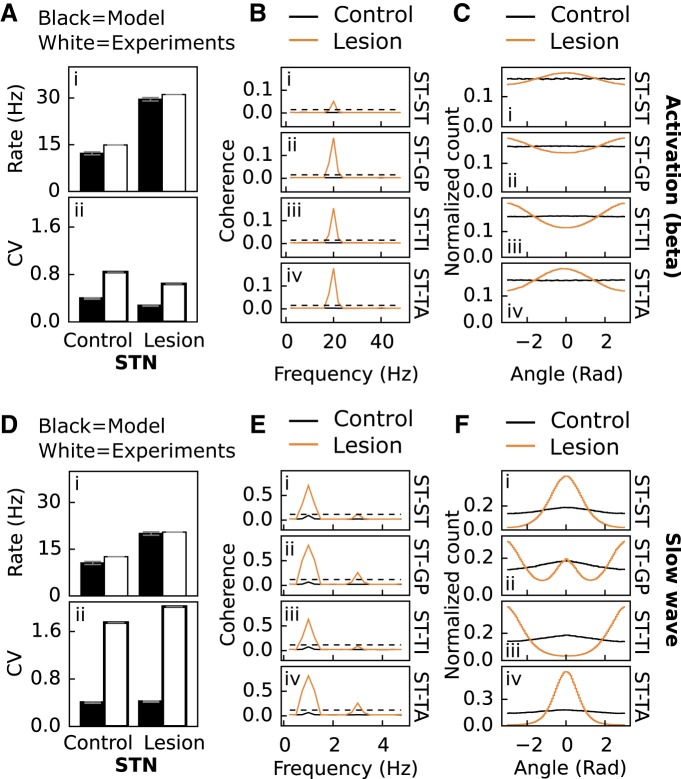
Model validation using STN as a readout. ***A***, Firing rate and CV of STN neurons with cortical beta input for model and experiment (Mallet, et al, 2008). ***i***, ***ii***, Firing rate and CV of STN neurons in control and lesioned (dopamine set to 0) network. ***B***, Coherence of STN and GPe neurons in control and lesioned network with cortical beta input. ***i–iv***, Coherence for STN versus STN, STN versus GPe, STN versus GPe TI, and STN versus GPe TA. Dotted black line shows significance of *p* = 0.05. ***C***, Phase relationship of STN and GPe neurons in control and lesioned network with cortical beta input. ***i–iv***, Phase relationship for STN versus STN, STN versus GPe, STN versus GPe TI, and STN versus GPe TA. ***D***, ***E***, and ***F***, same as ***A***, ***B***, and ***C***, for cortical slow-wave input.

In Mallet et al. (2008), the authors measured the spike statistics of GPe and STN neurons in rats under urethane anesthesia for two cortical states: during cortical activation, which was elicited by pinching the hindpaw, or during cortical slow-wave activity, which also resembles the activity observed during natural sleep. We found that GPe and STN firing rates for both cortical states could be reproduced by the model (Figs. 2*Ai*, *Di* and 3*Ai*, *Di*). The model could also capture the increase in CV of the GPe neurons between control and lesioned rats (Figs 2*Aiii, Diii*) for both cortical states as well as the decrease/increase in CV for STN neurons during activation (Fig. 3*Aii*). However, the weak increase in CV seen during slow-wave activity could not be reproduced (Fig. 3*Dii*).

The emergence of oscillations in BG and associated regions of the thalamus, caused by dopamine depletion, is a well-known phenomenon (Gatev et al., 2006). Mallet et al. (2008) showed significant coherence at 20 and 1 Hz for lesioned rats during both cortical states, both within and between GPe and STN neuron populations. The model could reproduce the coherence seen between randomly chosen GPe–GPe and between specifically chosen TI–TI, TI–TA, and TA–TA neurons (Figs. 2*B*, *E*) as well as between STN–STN, STN–GPe, STN–TI, and STN–TA neurons (Figs. 3*B*, E) for cortical activation and slow-wave activity in lesioned rats. After cortical activation in the control model, the coherence stays below the significance level (Figs. 2*B* and *3*B, black lines), whereas in the lesioned model the coherence between the pairs goes well above significance level (Figs. 2*B* and 3*B*, copper lines). Similar results were observed in simulations with cortical slow-wave activity (Figs. 2*E* and 3*E* )

The firing rate and CV relation of specifically the TA and TI neurons in lesioned rats found in Mallet et al. (2008) were also captured by the model. A puzzling observation that could prove difficult to reproduce by the model is that the firing rate of TI neurons are lower than TA neurons under cortical activation in dopamine-depleted rats (Fig. 2*Aii*), whereas under cortical slow-wave activity, the firing rate of TA neurons instead are higher than GPe TI neurons (Fig. 2*Dii*). It turns out that one solution to this puzzle had to do with the cortical and thalamic input to striatum, GPe and STN. We hypothesized that during cortical activation the input from cortex and thalamus is higher than during cortical slow-wave activity. Indeed, we found that hypothetically higher cortical and thalamic mean inputs during cortical activation than during cortical slow-wave activity could explain the TA and TI rate relation between the two cortical states (compare Fig. 2*Aii*, *Dii*). Also, the model reproduced qualitatively the observation in lesioned rats that the CVs of TI neurons were lower than CVs of TA neurons with cortical activation but then reversed with cortical slow-wave activity (compare Fig. 2*Aiv*, *Div*).

An interesting discovery in Mallet et al. (2008) was that TI and TA as well as STN and TI neurons in lesioned rats during both cortical states fired out of phase with each other, whereas STN and TA neurons fired in phase. We found that the model could reproduce this (TI–TA and STN–TI out of phase, Figs. 2*Ciii*, *Fiii* and 3*Ciii*, *Fiii*; STN-TA in phase, Fig. 3*Civ*, *Fiv*), and additionally that TI–TI, TA–TA, and STN–STN neurons in the model fire in phase during both cortical states (Fig. 2*Cii*, *Civ*, *Fii*, *Fiv* and 3*Ci*, *Fi*) as seen in Mallet et al. (2008). The model could also reproduce the single peak-valley coherence for GPe–GPe/STN–GPe neurons under cortical activation (Fig. 2*Ci* and 3*Cii*) and the double-valley coherence for the same pairs under cortical slow-wave activity (Fig. 2*Fi* and 3*Fii*). Thus the phase relationships for the GPe–STN network from Mallet et al. (2008) could be captured by the model.

### Mechanisms enhancing and quenching synchrony and oscillations in the dopamine-depleted state

**Figure 4.**
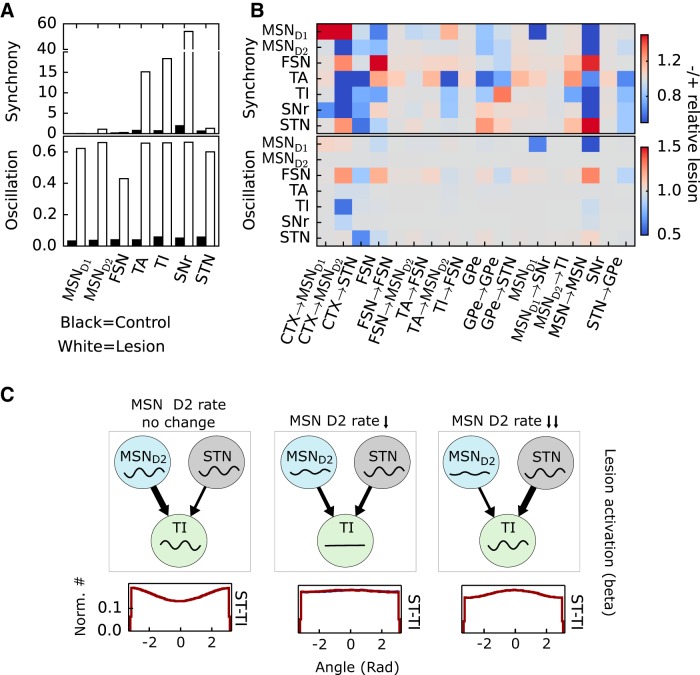
Local network dynamics and the role of dopamine-dependent perturbations. ***A***, Synchrony and oscillations in control and lesioned network components. Upper panel shows amount of synchrony in MSN D1, MSN D2, FSN, GPe TA, GPe TI, SNr, and STN during control (black bars) and after dopamine depletion (white bars). Lower panel shows the same but for oscillations instead of synchrony. ***B***, Upper panel shows the relative change in synchrony in MSN D1, MSN D2, FSN, GPe TA, GPe TI, SNr, and STN (*y* axis) compared with the lesioned network when restoring the parameter on the *x* axis to the value it had in the control network (no dopamine depletion). Lower panel shows the same but for oscillations instead of synchrony. ***C***, Phase relationship of STN and GPe TI when decreasing MSN D2 firing rate in the lesioned model. Cartoons illustrating effect of no change, medium decrease, and large decrease of MSN D2 firing rate. Signals represent instantaneous population firing rate in each nuclei.

The model exhibits appropriate enhancement of oscillations and synchrony in the dopamine-depleted state. Fig. 4*A* shows synchrony and oscillation index for all BG nuclei, with cortical activation as input for the control and lesioned (dopamine-depleted) models. It can be seen by comparing the black and white bars that there are significant increases in most of the indexes for different neuron populations.

It is clear from experiments that dopamine depletion has a significant effect on multiple network components in BG, e.g., by perturbing the synaptic signaling, connectivity degree, or neural excitability (Fig. 1*C*). However, the relative importance of each perturbation for driving the observed network changes after dopamine depletion (Mallet et al., 2008) is still not well understood. Thus to test how each individual perturbation affects the dynamics of the whole network, we ran several simulations. For each of the model parameters assumed to be perturbed after dopamine depletion, we restored the parameter values to the control, one at a time, and compared the simulation results with the model in which all parameters were set to the dopamine-depleted value.

We found that changes to neural excitability and synaptic coupling involving components of the GPe–STN network due to dopamine depletion have a diverse effect on synchrony and oscillations, with some neuronal populations showing increases and some decreases for a change in a single dopamine parameter. For example, restoring GPe excitability or lateral GPe connectivity both decrease synchrony in TA, but the TI effects are opposite (Fig. 4*B*). Restoring the dopamine depletion–induced increase in CTX to STN synapses has a dampening effect on synchrony in TA, TI, SNr, and STN and on oscillations in STN. Thus the reduction in excitability of GPe neurons or increase in coupling between CTX and STN seen in experiments after dopamine depletions has a significant effect on both synchrony and oscillations over multiple BG nuclei.

Restoring the perturbation on MSN D2–TI connectivity leads in our model to a decrease in synchrony in GPe TI neurons (Fig. 4*B*) in line with the results of Kumar et al. (2011). However, there is also a weak increase in synchrony for some other neuronal populations (Fig. 4*B*). Effects on oscillations are negligible.

Simulations suggested that restoring the perturbation to CTX–MSN D2 connectivity has a strong effect on synchrony and/or oscillations in BG with a decrease seen in MSN D2, SNr, TA, and TI (Fig. 4*B*) and an increase seen in MSN D1, FSN, and STN. A qualitatively similar effect in synchrony can also be seen when restoring the perturbation to MSN collaterals, except in MSN D1, where the result is the opposite.

We found it remarkable that restoring CTX–MSN D2 or MSN collaterals had such a dramatic effect on synchrony and/or oscillations throughout the network. Restoring both these perturbations decreases the firing rate in MSN D2 neurons. We hypothesize that oscillatory input from MSN D2 and STN competes in TI neurons. To test this, we ran three simulations with the lesioned network while varying the MSN firing rate. In Fig. 4*C*, it can be seen how the phase relation between STN–TI depends on MSN D2 firing rate, where a reduced firing rate of MSN D2 shifts the STN and TI neurons from out of phase, via cancelling each other, to in phase. In summary, the model results suggest that oscillatory cortical input conveyed via MSN D2 and STN competes in TI and that the effect can be balanced in TI. As the synchrony in TI goes down, TA and SNr also show less synchrony.

To summarize, we see that dopamine depletion–induced perturbations in general result in a dampening effect on synchrony and oscillations in some BG nuclei and a facilitating effect in others.

### Inhibitory control of MSNs and resulting effects for the striatal gating of cortical inputs

**Figure 5.**
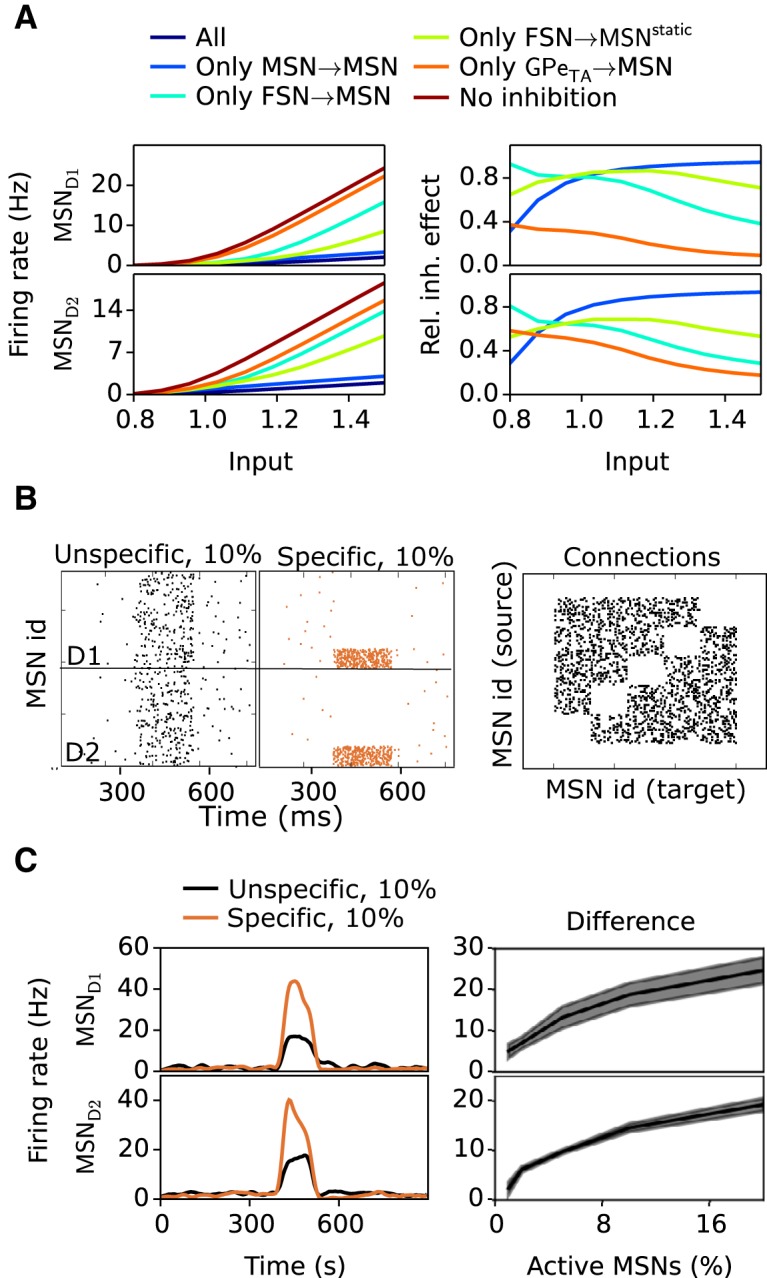
Control of firing rates of MSN neurons due to striatal inhibition. ***A***, Illustration how firing of MSN D1 and MSN D2 neurons are affected by inhibition from FSN, GPe TA, and MSN collaterals. Left top panel shows the firing rate of MSN D1 neurons with cortical input successively changed by a factor of 0.8 to 1.5 relative control, when keeping all inhibition (dark blue), only MSN–MSN inhibition (light blue), only FSN–MSN inhibition (cyan), only FSN–MSN static synapse inhibition (light green), only GPe TA–MSN inhibition (orange), and without any inhibition (dark red). Right top panel shows the relative contribution of the different sources of inhibition with successively increased cortical input and for MSN–MSN inhibition (light blue), FSN–MSN inhibition (cyan), FSN–MSN static synapse inhibition (light green), and GPe TA–MSN (orange) inhibition. Left and right bottom panels show the same as left and right top panels, respectively, except for MSN D2. ***B***, Illustration of the role of lateral inhibition when a subpopulation, 10%, of MSNs are activated. MSNs D1 and D2 are enumerated and given an index (id) between 1-1500. Left top panel shows the result when randomly selected MSN D1 neurons are activated; middle top panel shows the result when only nonconnected MSN D1 neurons are activated. Left and middle bottom panels show the same as left and middle top panels, except for MSN D2. Right panel shows the connection diagram for MSNs, in which a dot indicates a connection between MSNs. It illustrates how groups of MSNs, here those with low id, are not connected. ***C***, Left top panel shows the firing rate of the 10% of MSN D1 activated with a 100-ms-long input burst for the case when randomly connected MSNs (blue) and specifically nonconnected MSNs (red) are targeted. Right top panel shows the mean difference between the firing rate of the two differently selected populations of MSN D1 neurons when varying the activated MSN population size between 2% and 20%. Left and right bottom panels show the same thing as left and right top panels, except for MSN D2.

Inhibition from FSNs and GPe TA is important for controlling MSN firing rate at low cortical activity, whereas collaterals from neighboring MSNs may become important at higher cortical input (when MSNs spike more). The MSNs receive inhibitory input from three main sources, collaterals from neighboring MSNs, from FSNs, and GPe TA neurons. Earlier work has proposed that the weak, sparsely connected, and numerous MSN collaterals in the network set the overall excitability level (Wickens et al., 2007) and that strong feed-forward inhibition by FSNs acts as fast inhibition determining the moment-to-moment firing pattern of MSNs (Tepper et al., 2004). Thus two main players, collateral and feed-forward inhibition, have been considered important contributors to inhibition in striatum. But recently, a third player appeared, namely striatal inhibition from GPe (Mallet et al., 2012). Research showed that one GPe cell can form ∼10,000 synapses in striatum and that GPe synapses constitute a significant amount of the total number of inhibitory synapses in striatum. Here, we wonder what the role of inhibition from GPe is, and how it compares to collateral and feed-forward inhibition. To test this, we set up a simulation in which we increased the input from cortex to MSN, FSN, and STN in a stepwise fashion and measured the firing rate in a reduced striatal network with ∼3000 MSNs for five different scenarios when it comes to connectivity. The rest of the network neural populations (FSN, STN, GPe, and SNr) were also scaled down by a similar factor. We used ∼3000 MSNs, since that is the smallest striatal network in which MSN–MSN connection probabilities can be maintained. The result of Fig. 5*A* was also confirmed in the full network model of 80,000 neurons. We ran simulations with no inhibition onto MSNs, inhibition from MSNs only, from FSNs only, from GPe TA only, and finally with full inhibitory connectivity (Fig. 5*A*). It can be seen that FSN and GPe are more important in controlling the firing rate of MSNs during low cortical input, whereas MSNs are more important at higher cortical input (Fig. 5*A*). Thus the role of GPe seems to be more in line with FSN, which for low cortical input may control MSN firing, whereas collaterals control MSN firing rate relatively more at higher cortical input, thus acting as a regulator of MSNs overall excitability. Note that in the dopamine-depleted network, the relative importance of MSN–MSN inhibition will be changed, as MSN–MSN synapses are weakened while the inhibition from both FSN and TA rather increases.

### Could the intrastriatal lateral inhibition be important for the control of action selection?

As seen from the description above, activated MSNs that are not inhibited by collaterals from other active MSNs might have significantly higher firing rates. It has been proposed that the collateral network in striatum could work as a winner-take-all network (Wickens, 1997). For such a network to succeed, one has to assume strong connections between MSNs, but physiological evidence suggest that the connections are weak (Jaeger et al., 1994; Tunstall et al., 2002; Taverna et al., 2004; Tepper et al., 2004). To test whether weak connections between MSNs (0.15–0.45 nS) still can be important for controlling the local contrast between activated MSNs, a factor that could be important for action selection, we simulated two scenarios. We embedded in our reduced striatal network (∼3000 neurons) a number of nonconnected clusters of MSN neurons (Fig. 5*B*). In the first scenario, we randomly activated neurons from the whole pool of MSNs neurons, and in the second scenario, we specifically activated only nonconnected MSNs. It is clear that nonconnected MSNs spike higher than connected (Fig. 5*C*, first panel) and that this holds for over a range of different percentage of activated MSNs (Fig. 5*C*, second panel). Weak collateral MSN connectivity may thus be able to control the firing rate of neighboring MSNs and work as a mechanism increasing the contrast during action selection scenarios. This effect is expected to be significantly larger in a control network compared with a dopamine-depleted network.

### Further support for the action selection hypothesis

Kravitz et al. (2010) showed that MSN D1 activation promotes actions and MSN D2 activation inhibits actions. Cui et al. (2013) showed that both D1 and D2 MSNs are active in parallel during action selection experimental paradigms. These two studies suggest that populations of MSN D1 and MSN D2 that are activated by the same cortical activity state and are involved in the control of a resulting specific action do not project onto the same output neurons in SNr, since if they did, they would counteract each other based on the results of Kravitz et al. (2010). Therefore, we assume that the direct and indirect pathways converge on different SNr populations following co-activation by a specific cortical state that has been associated after learning with a certain action. To test whether the model supports the action selection hypothesis, we inserted two types of connectivity in the full model. The inserted connectivity, represented by activation of specific synapses between cortex and striatum, was assumed to have been learned from previous behavioral experiences. For models tackling this problem, see Potjans et al. (2011), Stewart et al. (2012), and Berthet et al. (2016). Strictly speaking, we tested with our model how suited BG are for transferring learned state action signals to motor-related output nuclei in thalamus or brainstem. However, this problem is not trivial. There is a high convergence of inhibitory MSN D1 connections on SNr (50:1), potentially resulting in a scenario in which SNr neurons are easily overwhelmed by striatal inputs and become unable to differentiate between MSN inputs representing different actions associated with certain cortical states. Below we first see how the model can behave when only action signals are communicating though the direct pathway.

**Figure 6.**
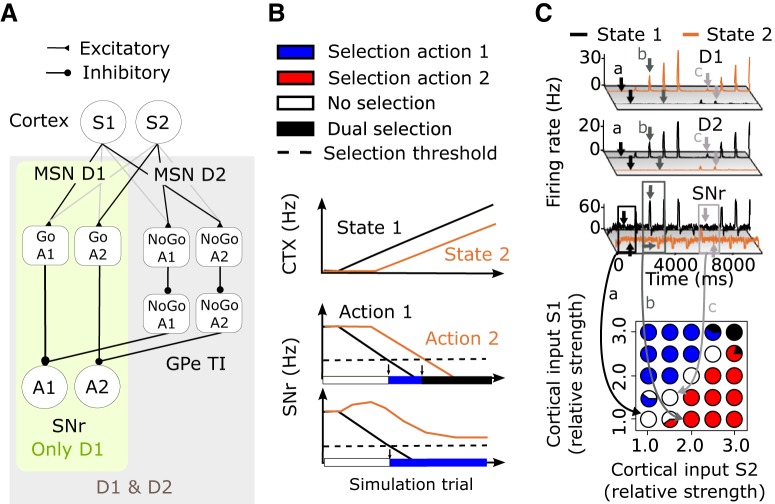
Illustration of the action selection network tested. ***A***, Illustration of a simplified action selection network with two action channels. Light gray lines from cortex to MSNs indicate that these connections are weak (e.g., due to learning), whereas black indicates strong connections. Cortical state S1 connects to one striatal population in the direct (Go) pathway (denoted Go_A1), which inhibits the action 1 (A1) population in SNr. S1 also activates a striatal population in the indirect (NoGo) pathway (denoted NoGo_A2), hypothesized to disinhibit SNr populations not compatible with action 1 (i.e. in this example action 2), and vice versa for cortical state S2. Green box indicates the network in which only the direct (Go) pathway is activated, and the larger gray box indicates the network in which both the direct (Go) and indirect (NoGo) pathways are activated. SNr A1 and A2 populations are in turn assumed to control targets in the brain stem and/or thalamus via disinhibition. ***B***, Illustration of two types of action selection scenarios. Top graph shows the firing rates of two hypothesized cortical states, state 1 (black) and state 2 (copper) that have been associated with two different actions after learning. Bottom two graphs show the resulting SNr firing rates for the co-activation of S1 and S2 representing action 1 (black) and 2 (copper). White indicates the interval on the *x* axis in which the SNr firing rates controlling action 1 and 2 are both above the selection threshold (i.e., no selection); blue indicates the interval in which the firing rate for action 1 is below the selection threshold, but action 2 is not (i.e., selection of action 1); and black indicates the interval in which both actions are selected (dual selection). In the beginning, good contrast can be obtained in SNr between the two actions (black and copper lines in middle panel), but at one point both will lead to completely inhibited populations in BG output nuclei, resulting in dual selection. Such a selection agent would work over a very limited range of inputs, and action selection would be possible only during a narrow window (middle panel, blue line). Instead, the capability to select only one action should preferably be obtained over a larger interval (bottom panel) with a mechanism ensuring that the contrast between the two actions is maintained over a larger range of inputs. ***C***, Illustration of how assumed cortical input combinations could activate striatal populations, in turn facilitating action 1 and action 2, and how illustrations of action selection outcomes are made. First and second panels show examples how D1 (Go) or D2 (NoGo) populations are activated for consecutive trials by cortical state 1 and 2. For example, state 2 are stepwise increased over five trials and state 1 is kept constant, and this is then repeated, e.g., five times but with state 1 increased each time. Third panel shows the resulting firing rate of SNr neurons representing action 1 (black) and action 2 (copper). Black, gray, and light-gray boxes are three examples of input combinations, a, b, c, as illustrated with the arrows and corresponding letters. Bottom panel illustrates how these types of simulations are classified into a selection pie chart. In each pie (circle) the relative proportions of action selection outcomes are represented with the respective colors: white for no selection, black for dual selection, blue for selection of action 1, and red for selection of action 2.

To test action selection capability, we set up two models. In the first model, “only D1,” we included two equally sized action pools in the MSN D1 network connected to corresponding action mappings in SNr (Fig. 6*A*). In the second model, “D1 and D2,” we also included action pools in the MSN D2 population that projected to corresponding pools in GPe TI, and finally the GPe TI projected to action mapping in SNr (Fig. 6*A*). It was assumed that the connectivity between cortex and striatum had been learned such that cortical state 1 (S1) activated a MSN D1 pool that in turn inhibited action 1 SNr neurons and at the same time activated MSN D2 neurons that increased the activity of action 2 SNr neurons (i.e., non–action 1 SNr neurons) through the indirect pathway. See Berthet et al. (2016) for how such connectivity can be learned. The opposite connectivity was assumed for state 2 when activating MSN D1 and D2 neurons. An underlying assumption is that there exist MSN D1 and D2 populations that represent a Go and a NoGo signal for a specific action, and that the corticostriatal connectivity is learned such that a certain cortical state activates one striatal Go population as well as one or perhaps several NoGo populations suppressing opposing actions. This is in line with the results of Kravitz et al. (2010) and Cui et al. (2013), in which a specific cortical state activates D1 and D2 MSNs that project onto different SNr neurons. In both the first and second models, we also could vary the sizes of the action pools in D1 and D2 neurons, e.g., between 10% and 100% of the corresponding neuron population type (see below). We thus formed what can be viewed as action channels through the BG (see Fig. 6*A*). Except for the assumed action channels, all other connectivities in the model were randomized.

Action selection should work over a range of different inputs and number of active presynaptic neurons. Humphries et al. (2006) showed how BG can perform action selection over a range of different activation rates of MSN neurons. Such properties are important for the dynamic range of the system. Imagine that we have two actions that are activated in a stepwise fashion at different firing rates, with one lower than the other (Fig. 6*B*). In the beginning, a good contrast can be obtained in SNr between the two actions (Fig. 6*B*, black and copper lines in middle panel), but at one point both will lead to completely inhibited populations in BG output nuclei, resulting in dual selection. Such a selection agent would work over a very limited dynamic range, and action selection would be possible only during a narrow window (Fig. 6*B*, middle panel, blue line). Instead, the contrast should preferably be obtained over a larger interval (Fig. 6*B*, bottom panel) with a mechanism ensuring that the contrast between the two actions is maintained over a larger range of inputs. In Humphries et al. (2006), the size of the activated MSN pool was not considered. Here we also propose that action selection should work over different proportions of activated MSNs. Thus it is important for the action selection agent to be able to select and gate through only one of the simulated cortical input states over a range of different input strengths and for various numbers of active presynaptic neurons.

To test the performance of action selection in our model, we measured the difference in firing rates between two groups of SNr neurons (action pools). We then generated a figure with the difference in firing rates between two action pools in SNr by presenting the network with a combination of cortical inputs leading to the activation with different strengths of striatal populations linked to actions 1 and 2 (Fig. 6*C*). Selection status was illustrated for each data point as a pie chart with no selection (white), selection action 1 (blue), selection action 2 (red), or dual selection (black). For each data point, we ran several simulations, and the proportions of outcomes are illustrated in each pie chart. The concept of selection threshold was adopted from Humphries et al. (2006), in which selection is considered to have occurred when firing rate becomes sufficiently low in SNr. Here the threshold is defined as 50% below base firing rate of SNr neurons (see Methods).

**Figure 7.**
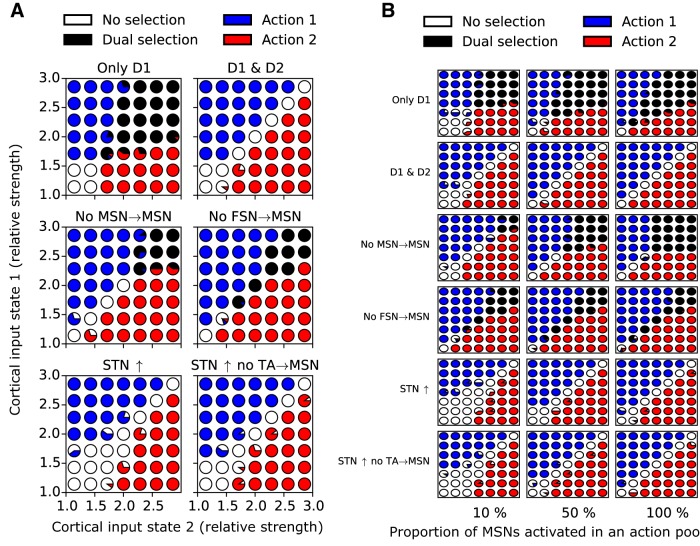
Action selection performance of the basal ganglia network. ***A***, Top left panel shows the action selection outcome with only the direct pathway activated, with respectively cortical input (i.e., S1 or S2) from 1× the base level to 3× the base level leading to selection of actions 1 and 2. Top right panel shows the action selection when both direct and indirect pathways are activated (similar action inputs as in top left panel). Middle left panel shows the action selection when both direct pathways are activated and MSN collaterals have been removed (similar inputs as in top right panel). Middle right panel shows the result when both direct and indirect pathway are activated and FSN–MSN inhibition has been removed (similar action inputs as in top right panel). Bottom left panel shows the action selection when both direct and indirect pathways are activated and in addition the hyperdirect pathway is co-activated through a burst in STN (similar action inputs as in top right panel). Bottom right panel shows the outcome when direct, indirect, and hyperdirect pathways are activated and GPe TA–MSN inhibition has been removed. For all six plots, 20% of the total MSN D1 or D2 pools were activated (the maximal MSN D1 or D2 action pool equaled each half of the total striatal MSNs). ***B***, Action selection performance when scaling the size of the activated MSN pool. First row shows the action selection with only direct pathway activated, same as top left figure in ***A***, but varying the relative size of the activated MSN population between 10% and 100%. Second row shows the same as the first row, except for the scenario when both direct and indirect pathways are activated. Third and fourth rows show the result for the scenario when both direct and indirect pathways are activated (as in row 2) and when in addition MSN collaterals or the FSN–MSN inhibition is removed. Fifth and sixth rows show the effect when the hyperdirect pathway is activated during action selection with and without TA–MSN present.

The indirect pathway is important for improving the dynamic range in the output, SNr, in response to cortical inputs to MSNs. The hypothesis that BG are involved in the selection of actions has been around for at least 20 years (Mink, 1996), but it is still debated to what extent BG are indeed involved (Nambu, 2008). Computational models have successfully been used to show that the idea is feasible (Gurney et al., 2001a; Humphries et al., 2006). These studies have made significant progress, stressing that the action selection hypothesis needs to be taken seriously. Here we have the chance to further test the action selection hypothesis within our quite quantitative model of the BG. The model has been built based on data collected from the literature with regard to activity levels, synaptic connectivity, and dopamine effects and validated based on known dynamics (i.e., not function) in the control and dopamine-depleted state; thus there is nothing that says that it should work as an action selection network. To test action selection capability, we started out with a model version with assumed action channels only in the direct pathway. It can be seen that the network performs action selection over a range of inputs (Fig. 7*A*, top left panel) but degenerates at higher inputs (black area), resulting in dual selection. Thus with only the direct pathway present, action selection can be performed, but only over a limited range of inputs. We then wanted to see what happened if we included the indirect pathway. Would it improve action selection such that dual selection is less likely? Indeed it did: adding action channels in the indirect pathway improves the contrast at higher input (Fig. 7*A*, top right panel). Finally we were interested in whether the model would perform action selection when varying the total size of the MSN pool assumed to be representing the action channel populations. In Fig. 7*A*, the D1 and D2 populations represent 20% of the total MSNs in the network, but interestingly, the effect persists when varying the number of active D1 and D2 neurons included into the action pools between 10% and 100% (Fig. 7*B*). Thus we found that the model supported the idea that BG can perform action selection, and that the indirect pathway is there to increase the dynamic range of the system by enabling action selection over a larger range of competing inputs.

### Inhibitory inputs to MSNs differentially affect action selection

Action selection deteriorates when MSN collaterals and/or feed-forward striatal inhibition are removed. Bronfeld et al. (2011) observed that when GABA-A is inhibited with bicuculline in the striatum, monkeys started to produce coarser and repetitive movements. They termed this loss of specificity (LOS). We were now interested in whether the model could give an explanation why inhibiting GABA-A produced LOS. To test the role of inhibition, we blocked GABA synaptic projections, one type at a time, from MSN collaterals and FSN. We found that when removing the inhibition onto MSNs from either collaterals or the FSNs, the model showed evidence of LOS. For both scenarios, action selection gets worse with dual selection when combining two strong inputs (Fig. 7*A*, middle panels). The model thus predicts that both MSN collaterals and FSNs are important for robust action selection in BG. This observation also holds when a different proportion of the MSN pool is activated (Fig. 7*B*).

### STN as a transient stop signal

STN, through the cortical hyperdirect pathway, is proposed to act as a stop signal (Gillies and Willshaw, 1998; Frank, 2006), giving striatum and GPe enough time to resolve high conflict in action selection situations. Experiments also suggest that STN is involved in the cancellation of already initiated motor responses (Eagle and Robbins, 2003; Eagle et al., 2008; Schmidt et al., 2013, Mallet et al. 2016). We ran simulations in which we added input to STN as a 100-ms pulse (to match the duration of the striatal activation seen in experiments) in parallel with activation of the striatal action pools (Fig. 7*A*, bottom left panel). We observed that action selection at low cortical input was stopped or delayed, in line with the claim that STN can be responsible for a transient stop signal in the BG (see also Lindahl et al., 2013). When GPe TA projections to MSNs were removed, the stop signaling via STN was weakened. This is in agreement with suggestions made in Mallet et al. (2016) that the activity in MSNs could be decreased by activity in the TA–MSN inhibitory synapses (also compare Fig. 7B, bottom two panels).

### Action selection in the dopamine-depleted network

**Figure 8.**
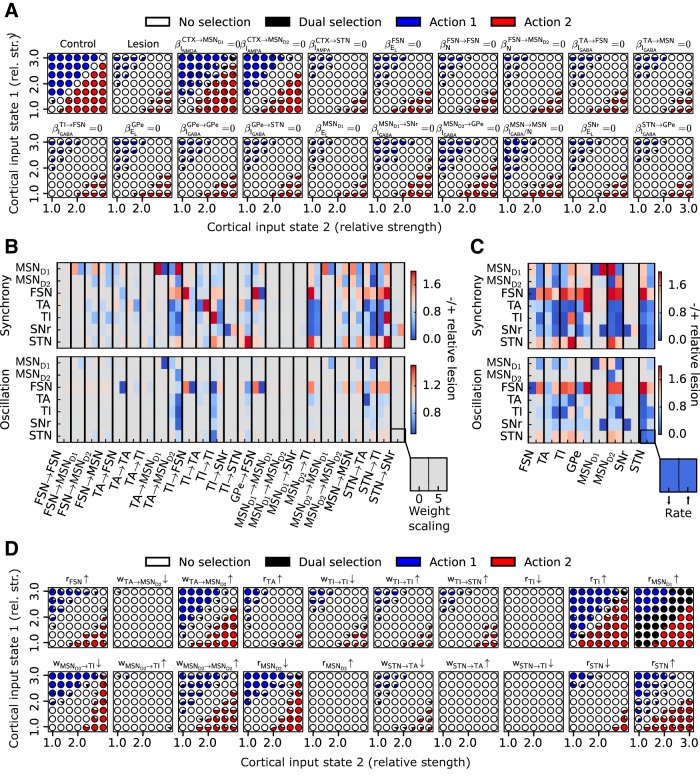
Restoring network dynamics and function after dopamine lesion. ***A***, Action selection performance of control and lesioned networks. Left upper panel shows an action selection heat map of the control network with direct and indirect pathways both activated. Second upper panel shows the same but for the lesioned network. The remaining panels show the result for the lesioned network but with the dopamine parameter displayed in the heading restored to the control network parameter value. (compare with Fig. 4*B*). ***B***, Change in synchrony and oscillations in MSN D1, MSN D2, FSN, GPe TA, GPe TI, SNr, and STN relative to the lesioned network model when silencing/increasing each connection. ***C***, Change in synchrony and oscillations in MSN D1, MSN D2, FSN, GPe TA, GPe TI, SNr, and STN relative to lesioned network model when inhibiting/exciting each nucleus separately. A current was injected with a negative or positive value in each nucleus. The hyperpolarizing and depolarizing current steps were equally large, where the amplitude of the hyperpolarizing current was adjusted to silence the specific nucleus (mimicking a lesion of the nucleus). ***D***, Action selection performance for a number of manipulations either to neuron excitability or to synaptic efficacy.

Action selection deteriorates when dopamine is removed from the network. Dopamine loss leads to increase in synchrony and oscillations in BG as seen above, but it is not clear whether dopamine loss would also affect action selection in our model. Actually the pathophysiology of PD in BG is still not well understood (Nambu, 2008). The firing rate model by DeLong (1990) proposed that imbalance between the direct and indirect pathways changes the mean firing rate of BG output nuclei and induces PD. The firing pattern model by Bergman et al. (1998) instead suggests that it is the increase in oscillations that interfere with BG information processing in PD. It is not easy to resolve whether it is the firing rate or firing pattern model that better explains the pathophysiology behind PD. The answer is probably both. We wanted to see whether our model could shed light on this controversy. Without dopamine, the model network is not able to efficiently perform action selection (Fig. 8*A*, top second panel). Dopamine changes the balance between the direct and indirect pathways, such that the indirect pathway becomes more dominant, leading to action selection failure and an increased firing rate in the SNr. Thus the model supports the assumption that excessive firing in the output of BG can underlie some of the symptoms in PD.

### Reversal of selected dopamine-depletion induced network changes restores action selection

Dopamine depletion induced action selection deterioration, but this can be restored in the model by reverting the dopamine-induced change in mainly CTX–MSN D1 and CTX–MSN D2 synapses (Fig. 8*A*), despite still having synchrony or oscillations present (compare Fig. 4).

### Effects of synaptic increases or decreases on oscillations and BG action selection capability

Several perturbations to the connections in BG lead to a decrease in oscillations, and in some cases also improved action selection, thus making them potential targets for animal models or translational studies. For example, in Fig. 4, it was shown that reverting just one parameter at a time had effects on the synchronizations and oscillations in the dopamine-depleted network. To further test how parameter perturbations could affect both oscillations and action selection, we set up simulations in which, on top of beta oscillations from cortex in the lesioned model, we added the test of action selection. Action selections were measured as a certain decrease in mean firing rate of SNr neurons during the action selection phase (see Methods). It was found that decreasing or increasing the synaptic efficacy in approximately half (11/25) of the modeled connections led to a decrease in synchrony or oscillations, and that in a subset of them the change (i.e., increased connectivity for TA–MSN D2 and MSN D2–MSN D2 and removing connectivity for MSN D2–TI) also improved action selection ability. Table 10 lists the connections with either decreased or increased efficacy that in Fig. 8*B* show significant reduction of synchrony or oscillations in several nuclei. All manipulations of connections in Table 10 could thus serve as potential targets for animal models or translational studies. Especially interesting are the manipulations that also improve action selection (Fig. 8*D*).

**Table 10.** Effect of connection on synchrony (+, increase; –, decrease, 0, no change), oscillations (+/–/0), and action selection

Connection	Scaling	TA	TI	SNr	STN	Action selection
TA-MSN D2	0	+/0	+/0	+/0	0/0	Worse
TA-MSN D2	5	+/+	+/+	+/+	0/0	Improved
TI-TI	0	0/+	–/+	0/+	–/+	None
TI- TI	5	+/0	+/0	+/0	0/0	None
TI-STN	5	+/0	+/0	+/0	–/0	None
MSN D2-TI	0	+/+	+/0	+/0	–/0	Improved
MSN D2-TI	5	+/0	+/0	+/0	0/0	Worse
MSN D2-MSN D2	5	0/0	+/0	0/0	–/0	Improved
STN-TA	0	+/+	0/0	0/0	0/0	None
STN-TA	5	+/0	+/0	+/0	–/0	Worse
STN-TI	0	+/0	+/0	+/0	0/0	Worse

### Effects of nuclei lesion and stimulation on oscillations and BG action selection capability

By silencing or increasing the activity of specific BG nuclei, an improvement in action selection is achieved (Table 11 and Fig. 8*C*, *D*). Lesioning GPe and STN decreases both the synchrony and oscillations in several nuclei in the network, lesioning is especially effective in STN (Fig. 8*C*). Both predictions are in accordance with experiments. Lesion therapies targeting GPe, GPi, and STN have successfully been used to alleviate PD symptoms (Okun and Vitek, 2004). It can also be seen that increases in the activity in STN leads to a decrease in synchrony and oscillations, which is again in line with what deep brain stimulation (DBS) in STN has proven to be an effective method for relieving PD symptoms.

**Table 11.** Effect of nucleus firing rate on synchrony (+, increase; –, decrease; 0, no change), oscillations (+/–/0), and action selection

Nucleus	Firing rate	TA	TI	SNr	STN	Action selection
FSN	Increase	+/0	+/0	+/+	–/0	Improved
TA	Increase	+/+	+/+	+/+	–/–	Worse
TI	Silent	+/+	+/+	+/0	–/0	Worse
TI	Increase	+/+	–/0	+/+	–/–	Improved
MSN D1	Increase	0/0	0/0	+/+	0/0	Improved
MSN D2	Silent	+/+	+/+	+/+	–/–	Improved
MSN D2	Increase	+/+	+/+	+/+	–/–	Worse
STN	Silent	+/+	+/+	+/+	+/+	Worse
STN	Increase	+/0	+/+	+/0	+/+	Improved

The model holds one possible answer to the paradox that either decreased activity in STN (by lesioning it) or increased activity in STN (through high-frequency stimulation) can reduce pathological dynamics. When STN is lesioned in the model, TA and TI neurons dramatically decrease in firing rate and no longer convey oscillations to SNr neurons. On the other hand, when STN firing rate is increased, TA firing rate is elevated, in turn reducing MSN firing rate. This shifts the balance between oscillatory inputs from cortex conveyed via MSN D2 and STN onto TI neurons (compare Fig. 4*C*).

Both decreasing and increasing MSN D2 activity counteracts synchrony and oscillations in GPe and SNr. Loss of dopamine leads to increasing activity in MSN D2 neurons. Decreasing MSN D2 activity (Fig. 8*C*) proves to be an effective way to annihilate synchrony and oscillations in GPe and SNr. On the other hand, increasing MSN D2 activity silences GPe, resulting in the same output as lesioning GPe. However, only the decrease in MSN D2 activity is predicted to improve action selection capability (Fig. 8*D*).

### Model robustness: some observations

The BG model was built on current data and knowledge, but several parameters are unknown, especially with regard to the synaptic connectivity patterns between different nuclei. During the process of validating the model against Mallet et al (2008) additional insights were received regarding parameter constraints. These findings are described below.

**Figure 9.**
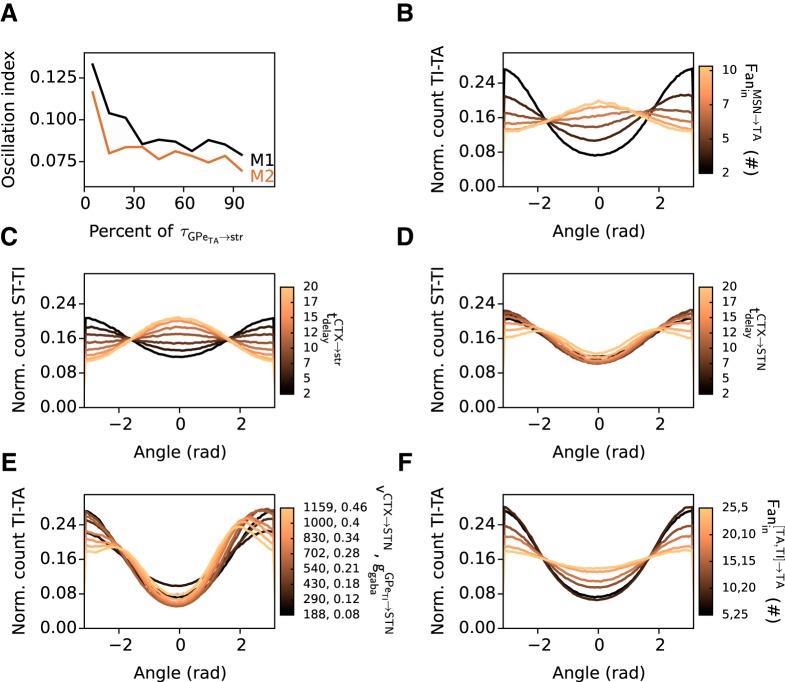
Effect of network parameter perturbations. ***A***, Effect of synaptic decay $\tau_{GPe_{TA}\to \text{striatum}}$ on oscillations in MSNs in the control network. Black line shows effect on MSN D1 and copper line shows effect on MSN D2. ***B***, Phase relation between TI and TA neurons when fan-in to TA neurons from MSN was varied between 25 and 125 with step 25. ***C***, Phase relation between STN and TI neurons when varying the delay between cortex and striatum from 2.5 to 20 ms with steps of 2.5 ms. ***D***, Phase relation between STN and TI neurons when varying the synaptic delay between cortex and STN from 2.5 to 20 ms with steps of 2.5 ms. ***E***, Phase relation between TI and TA neurons when keeping STN activity constant by varying the synaptic weight between TI and STN in parallel with adjusting the background input rate from cortex to STN. ***F***, Phase relation between TI and TA neurons when fan-in to TA neurons from TA and TI neurons were, respectively, [25,5], [20,10], [15,15], [10,20], and [5,25].

One observation was that synaptic dynamics of TA to MSN could control oscillations in striatum. The dynamics of the GABAergic TA–MSN synapse prevents oscillations in striatum under normal dopamine conditions. The time constant that Glajch et al. (2016) recorded for TA synapses on MSNs is >6 times longer than a normal GABA synapse (10 ms vs. 60–90 ms). Is there a reason this is important? It turned out that this time constant was not crucial in explaining Mallet et al. (2008), but instead oscillations in the MSN populations started to occur in the control model with normal dopamine levels when the time constant was decreased too much (Fig. 9A). Thus the simulations predict that the time constant of the TA–MSN synapse indeed needs to be significantly slower than the other GABA synapses to prevent oscillations in MSNs.

A second insight gained was that MSN–TA connectivity can affect the phase relation and even can reverse the phase relation between TI and TA in the model with dopamine depletion. Chuhma et al. (2011) showed that there is a population of GPe neurons that do not receive input from MSN neurons. They showed that 28% of the GPe neurons in their study were not activated by light stimulation of striatal fibers. In the current study, we assumed that TA neurons corresponded to this population. One reason was that it made sense, since TA neurons fire in phase with STN, implying that they are controlled by excitatory input from STN rather than inhibitory inputs from MSNs. We wanted to challenge this assumption. MSN–TA synapses were added to the model with conductance similar to that of MSN–TI synapses. The model shows that even with an MSN–TA fan-in (number of incoming of connections) at 25 (compared with MSN–TI at 500), the phase relation of TI and TA was significantly altered (Fig. 9*B*). With fan-in at 100, the phase relation starts to reverse where TI and TA neurons now are in phase with each other. Thus the model supports the assumption that TA indeed receives none or only a low number of connections from MSNs.

A third insight was that the synaptic delay between cortex and striatum/STN is important. Synaptic delays between cortex and striatum/STN affect the phase relation between STN and TI neurons. Cortical oscillations can affect TA neurons though two main pathways, one inhibitory through cortex to MSNs and one excitatory through cortex and STN. Mallet et al. (2008) showed that STN and TI neurons fire out of phase in lesioned rats. It was crucial that the model could reproduce this dynamic, and we hypothesized that the delay between cortex and striatum/STN could play a crucial role in enabling us to reproduce the dynamic. We found that stepwise increasing the delay from 2.5 up to 20 ms for cortex to striatum could distort the phase relation between STN and TI neurons (Fig. 9*C*), and the same holds for cortex/STN (Fig. 9*D*). Thus the phase relation of STN and TI neurons is likely governed by the corticostriatal/STN delays, and this suggests that these parameters need to be controlled well in the real system.

We also tested the role of increased connectivity between STN and GPe for TI–TA phase relation. The model does not support stronger GPe–STN synapses than those we chose to have. Farries et al. (2010) observed that when removing GPe connections to STN, the firing rate of STN neurons doubles and the cortical input to STN and GPe–STN conductance were originally tuned to reproduce this result (compare Lindahl et al., 2013). However, another study (Féger and Robledo, 1991) reported that STN firing rate increases fivefold when removing input to STN. Moreover, Terman et al. (2002) showed that strengthening of the GPe–STN synapses leads to oscillations in GPe and STN neurons. Thus we hypothesized that GPe–STN synapses were stronger. Therefore we modified GPe–STN synapses and input to STN neurons such that removing GPe neurons increased STN firing rate from 20 up to 50 Hz with 5**-**Hz steps. We found that strengthening GPe–STN could not be supported by the model, since it introduces a double peak in the phase relation of TI and TA neurons (Fig. 9*E*). Additionally (not shown), the firing rate of TI and TA in the lesioned model increases a lot and no longer compares to Mallet et al. (2008).

The model also suggests that the input from TI and TA onto TA preferably comes from TI neurons rather than TA neurons. It is known that GPe neurons receive collateral input from surrounding GPe neurons. What is not known is how TI and TA neurons connect to each other. Because TI neurons send significantly more collaterals than TA neurons (Mallet et al., 2012) and TI neurons are five times more numerous, then by share numbers TI neurons should receive most of their collaterals from TI neurons. A similar argument could be made for TA neurons; that is, they should receive most of the collaterals from TI. But this may not be true, so we ran five simulations in which the fan-in from TI and TA neurons to TA neurons was altered. The phase relation between TI and TA neurons is weakest when the majority of inputs to TA are from TA and strongest when the majority comes from TI neurons (Fig. 9*F*). Thus the model predicts that TA neurons preferably receive input from other TI neurons.

## Discussion

We investigate in a spiking network model of BG the mechanisms for both the control of oscillations and spike synchronization during dopamine-depleted stages and further correlate the network dynamics to how action selection is supported. We explore how different sources of inhibition in striatum contribute to these network phenomena and hypothesized function. Our model was built using parameters compiled from a large set of experimental data sets, and the subsequent validation of the model against multiple experimental observations aims at making it a quantitative model of the BG system that can be used to make predictions and help increase our understanding of the mechanisms behind action selection and dynamic features occurring during PD, and in the future function as a framework for incorporating biophysically detailed model modules of various neuron types or even whole nuclei. During the model building and model validation process, we explored the effect of several model parameters. We predict that inhibition from FSN and GPe in striatum is relatively more important during low cortical input, whereas the collateral MSN network becomes more significant during higher cortical input. It is also demonstrated by implanting a topology in the weak collateral MSN network that the contrast between input signals can be enhanced, which could facilitate the action selection capability. The indirect pathway is predicted to increase the dynamic range of action selection signaling in the BG, where action selection is preserved also for stronger cortical inputs, a result in line with Humphries et al. (2006). The feed-forward and collateral inhibition in the striatum is also predicted to be important for increasing the dynamic range. It is also demonstrated that a brief, high input from STN can stop or delay action selection, similar to what Frank (2006) predicts, and here the GPe TA to MSN inhibition contributes in line with Mallet et al. (2016). Dopamine depletion in the network leads to an increase in spike synchrony and oscillations, and at the same time an impairment in action selection capability is seen. Our simulations predict that an important mechanism behind these changes in network dynamics and BG function is the increase in the activation of the CTX–MSN D2 pathway, the weakening in the MSN collateral inhibitory network, and to some extent the decreased excitability of GPe neurons after dopamine depletion. It is shown that when successively manipulating the excitability in each of the BG nuclei, either increasing or decreasing their excitability, a reduction in the synchronization and the oscillations as well as an improvement in action selection can be achieved. We also found that either a decrease or an increase in the connectivity of at least 10 different connections in the BG network can decrease the synchrony and oscillations in the dopamine-depleted state. For about half of these changes in the connectivity the action selection capability is improved.

The effective connectivity used in this model compares well to the predicted connectivity from a rate model by Nevado-Holgado et al. (2014) that was fitted to part of the same data set. They similarly predicted that the connectivity between MSN–GPe TI is strong, whereas MSN–GPe TA projections are weak. It is also shown here that TI–TA should be stronger than TA–TA, thus implying that TI neurons have a strong control over TA neurons. Both these findings also seem to be supported by experiments (Mallet et al., 2012). However Nevado-Holgado also predicted that STN connectivity is stronger to TA than to TI. This is something we did not find; actually the conductance of STN–TA synapses in our model is ∼3 times weaker than STN–GPe TI connections. However, we still saw that STN influences the activity in GPe TA in practice, since the STN input constitutes a larger proportion of the total excitatory input to GPe TA compared with the total excitatory input to GPe TI. Thus the resulting effective connectivity in our spiking network model is still in line with the predicted (relative) connectivity used in the rate model by Nevado-Holgado et al. (2014).

BG modeling has made significant progress in proposing how the architecture of BG supports the hypothesis that BG are a general-purpose action selection device (Gurney et al., 2001a,b; Humphries et al., 2006). For example, the spiking network model of Humphries et al. (2006) contained detailed BG physiology and could replicate multiple experimental data sets. Since then, new data on synapses and connectivity have been collected and need to be included in BG models. Thus in this study, we extended the functional connectivity of previous models (e.g., Humphries et al., 2006) with short-term plasticity, used a more detailed striatal network, took into account the relative BG nuclei sizes, and also included the GPe TA/TI populations. Similar to Humphries et al. (2006), we found that BG can support an action selection function.

Our spiking BG network model provides novel insights about action selection, synchrony, and oscillation-quenching mechanisms and provides a framework for investigating PD and other BG-related diseases. The current model, however, lacks the thalamic and cortical feedback loops from the BG output nuclei to influence the BG input via one longer thalamus–cortex–striatal loop and a shorter thalamus–striatal loop (Smith et al., 2010). Spike synchrony, network oscillations, and BG action selection functionality could very well be affected by these loops. Although implementing the longer loop via cortex would be a major project, including the shorter loop would be a natural next step.

## Synthesis

Reviewing Editor: Marlene Bartos, University of Freiburg Decisions are customarily a result of the Reviewing Editor and the peer reviewers coming together and discussing their recommendations until a consensus is reached. When revisions are invited, a fact-based synthesis statement explaining their decision and outlining what is needed to prepare a revision will be listed below. The following reviewer(s) agreed to reveal their identity: Mark Humphries, Nicolas Mallet

This manuscript is a computational study based on spiking model with a special focus on the question of how different sources of striatal inhibition impact neuronal network dynamic including network oscillations, synchrony and action selection. The model includes realistic connectivity patterns and most parameters included in the model have been extracted from experimental datasets. All reviewers judged the study as nice and interesting. However, they encourage the authors to pay more attention to the quality of their writing as the paper contains many typos, unclear statements and errors that impact negatively on the overall quality of the work. See the major comments and criticism below.

Reviewer 1:

The paper reports an extensive investigation of both the model's dynamics and its potential insights into basal ganglia function. It is able to replicate complex data on the pattern of oscillations within the basal ganglia and their changes due to dopamine depletion, across multiple cortical states. Compellingly, the authors use their model to identify multiple potential key contributors to pathological dynamics in Parkinson's disease, thus suggesting targets for animal model and translational studies. Finally, the model offers a potential resolution to the paradox of the equal effectiveness of DBS and lesion surgery for Parkinson's disease. The authors are to be congratulated on an exceptional achievement.

The quality of the science is self-evident, and deserves publication. My comments are thus concerned only with improving the clarity of the manuscript, to make it more widely-accessible and to draw out the insights from the model's results.

Comments:

(1) The “whys”. The model shows a range of interesting dynamical phenomena. But often the insight into how these arise from the model is lacking - the advantage of a model is that we have some chance of understanding *why* a particular effect occurred. Could the authors please address at least these results of most outstanding interest:

(i) Lines 647-651: why does restoring the cortex->MSND2 connection to its healthy value have such a dramatic effect on both oscillation and synchrony throughout the basal ganglia model?

(ii) Lines 805-813. The model shows that either lesioning the STN, or over-activating it, can reduce oscillations and synchrony throughout the basal ganglia. This seems to offer a solution to the long-standing paradox of why both DBS and lesion interventions in Parkinson's are equally effective. So please make this conclusion, and give some insight into why the model predicts this effect! [Also, perhaps point out a unique strength of this model: that altering the STN can directly reduce synchrony and oscillations in the MSNs and FSNs - thus the model clearly predicts that its STN-GP-striatum pathway is sufficient for STN manipulation to affect the whole of the basal ganglia]

(2) Network model (lines 117-121): a “one-dimensional representation was used” meaning the MSNs and FSNs were placed on a line (i.e. a single“layer”)? Randomly mingled? How did this then map to the other nuclei?

(3) Model input (lines 133-139). Many details are missing here:

(i) How were the slow-wave and “activated” input states modelled?

(ii) What is parameter “a” in Table 1?

(iii) How were the inputs during the action selection simulations generated - as a Poisson process?

(iv) The text says “The length of the burst was set to 100ms...”. What burst? When did these occur during the simulation, and in what simulations?

(4) Lines 484-499. The text claims the model reproduces the increase in STN CV between control and lesion states during slow-wave input. But Fig3C shows no difference in the model CV values between these states. Please clarify

(5) The section “Mechanisms enhancing and quenching synchrony and oscillations in the dopamine depleted state” is both too long and very hard to follow. The logic of the work needs to be made clearer: first the model shows appropriate enhanced oscillations and synchrony following DA depletion (Figure 4A); second, each DA perturbation was returned to its healthy state in turn, to understand its contribution to the changes in oscillation and synchrony. The text then needs to ensure that all simulations are framed in that way - which currently they are not consistently (e.g. lines 632-634).

(6) Section “Inhibitory control of MSNs is dependent on activation level” is not clear about what model was used. The Methods says that a striatal network of 3000 neurons was used here; but the Results text indicates that input to the STN was part of the simulations. So was this a full BG model, scaled down? If so, why? How was GP input modelled if it was not a full BG model?

(7)Section “Support for the action selection hypothesis” is not clear on how the model was set up to test action selection. Was this a full BG model, or just the sub-set of nuclei in Figure6A? If a full model, how were the actions representing populations defined in STN and GPe-TA?

(8) Figures 6-8: all action selection tests show pie-charts, but do not explain what these pie-charts are. They seem to show the proportion of tests in which each of the 4 possible selection outcomes occurs for a given pair of inputs; but the Methods and Results describe only testing 25 different combinations of inputs from S1 and S2, not repeated tests of the same inputs. Please explain

(9) Section “Effect of synaptic increase or decrease on oscillations and action selection” needs an improved text:

(i) Why do this? Motivate the work - presumably the search here is for useful insights into potential treatment targets

(ii) How are oscillation and synchrony measured during the action selection experiments? Between the stepped inputs (cf Figure 6 panel C)?

(iii) And if there is oscillation happening during action selection, how does one then define action selection as a crossing of a fixed threshold?

(10) Figure 9 and all accompanying main text (lines 820 onwards) appears to be in the wrong place: these are all robustness results for the GP and STN changes between healthy and lesioned states. Surely these should be placed directly after Figure 3?

Minor text issues:

(i) Line 266: Bolam et al 2000 is a review; perhaps Bevan et al 1998 J Neurosci was meant here?

(ii) Lines 287-292: the numbers used here seem to have some typos: e.g. line 287, 10 multiplied by 1000 does not equal 9000! Check and fix

(iii) Lines 294-296: I could not understand the sentence “Thus for each TA connections we have 16 TI onto a GPe cells when accounting for nuclei size as an estimate based on neural and connection count”. Which GPe cells? What does “accounting for nuclei size” mean here?

(iv) Lines 340-344. If the AMPA:NMDA conductance ratio is 2:1, then why is the actual conductance ratio used an order of magnitude larger than this? (~26:1, given 0.5nS for AMPA, and 0.019nS from NMDA)

(v) Lines 364-365: “assuming balances input output STN input to GPe TA was also lowered”???

(vi) Lines 374-375: presumably alpha_dop, not alpha_0, here?

(vii)Eq 7: what are the mean and variance of? One presumes spike counts in some bin? And if so, what bin size?

(viii) Eq 8: What is the power spectrum S computed from? The rate histogram of the population? An average over the spectra of individual neuron spike-trains?

(ix) Lines 482-483, and thereafter: the “CV” of what? Inter-spike intervals presumably?

(x) Lines 467-564: many of the references to the figures refer to the wrong panels - please fix (e.g. line 524 says “Figure 2B top left panel” but seems to be referring to “Figure 2C top right panel”).

(xi) Lines 527-530. Issues here of data interpretation. The text says “We hypothesised that in active rats the input from cortex and thalamus are higher than in resting rats”. All data from Mallet et al 2008 is under urethane anaesthesia, and so there are not “active” or “resting” rats, but two different cortical states under anaesthesia (as the authors elsewhere use correctly - slow-wave and “activated” by a hindpaw pinch). Again this seems to be an issue stemming from the lack of description in the model inputs: these two states are of course underpinned by very different firing of cortical and thalamic cells - the only question is, how were these modelled?

(xii) Lines 749: “15-100 percent” of what? The action pool? How big was each action pool relative to the size of the modelled nuclei?

(xiii) Lines 765-766. The hypothesis that the STN is a “stop” or “braking” signal is due to Gillies & Willshaw (1998) “A massively connected subthalamic nucleus leads to the generation of widespread pulses” Proceedings of the Royal Society of London-B, 265, 2101-2109.

(xiv) Lines 769. Why add STN input as a 100ms pulse? What is the motivation for that short burst?

(xv) Line 788 “Dopamine depletion induced action signal inference” ??

(xvi) Lines 802-819: help the reader here by pointing out which activation changes improved both action selection and reduced oscillations/synchrony

(xvii) Line 827; what is the “control model”?

(xviii) Line 836: what is the “lesioned model”?

(xix) Lines 860 - 910: consistently refers to the wrong panels in Figure 9.

Reviewer 2:

1/ The first paragraph of the result section dealing with the validation of the model is lengthy and difficult to read. Could this part be shortened?

2/ Result section 'Mechanisms enhancing and quenching synchrony and oscillations in the dopamine depleted state': It is difficult to make the parallel between the text explanation (in the result section) and the change in synchrony and oscillations in figure 4B. It would be nice to add additional numbers on top of the figures to help make this link.

3/Methods line 201: The firing rate if type A neurons are considerable lower than type B (4 compared to 19 Hz..)'. In fact the number are closer to 5 vs. 20 Hz according to the original reference.

4/ GPe neuron model: The authors includes external excitatory inputs from thalamus to GPe-Ta and Ti to account for the difference in their firing rate. It is not clear here why the authors choose excitatory inputs coming from thalamus rather than elsewhere (i.e. STN) ? Additionally, which part of the thalamus the authors are referring to? A citation would also be helpful to readers.

5/ MSN neuron models: the authors mention that the effect of dopamine D1 receptors activation induces both hyperpolarization (through KIR) and increase of neural response but it is not clear to me what would be the net effect of D1 receptor activation (excitation or inhibition?) with an α0 = 0.8 (representing normal dopamine transmission, see line 171). Also, in the MSN neuron model description there is no mention of the effect of dopamine on D2 MSNs? How is this modeled?

6/ In the Connectivity section: The assumption that Ti neurons do not project back to striatum is incorrect! Indeed, it has been clearly shown that a subpopulation of GP neurons projecting to STN and SNR also sent inputs to striatum and selectively targeted GABAergic interneurons (see Bevan et al., 1998; Saunders et al., 2016). Why did the authors omitted this well-known connection here? In fact, it would have been nice to investigate the consequence of such connectivity pattern to help understand its functional importance in controlling MSNs activity. Similarly, in the section 'Inhibitory control of MSNs is dependent on activation level'. According to the authors, the role of GP-Ta inhibition seems to be more in line with the role of FSN during low cortical input. Because of the interplay between GP-Ta and GP-Ti, and the fact that GP-Ti neurons selectively target FSNs, it would have been equally interesting to study the impact of the omitted connectivity on the network dynamic.

7/ line 692: Freeze et al., (2013) showed that both D1 and D2 are active during action selection suggesting...'. However, a better reference for such statement would be the work from Cui et al., 2013 Nature DIO: 10.1038/nature11846.

8/ Schematic diagram in 6A in confusing with regards to the authors statement line 696: 'we assume that direct and indirect pathways..' (presumably belonging to the same actions) '..converge on different SNr populations..'. The figure 6A is correct when considering the cortical inputs S1 and S2 but it is confusing to show that D1 and D2 neurons belonging to the same action (i.e. A1) converge onto the same SNr neuron. Please amend the figure accordingly.

9/ What is the unity scale of x and y axis plots in Fig 6C (second panel),and Fig 7 A and B ?

10/The action-selection capability of the authors' model can be argued if the cortical inputs show already a selectivity of one action as compared to the other. Indeed, if the cortical inputs to striatum are already biased toward one action then the model is not strictly speaking performing action-selection but rather reinforce the already selected (at the cortical level) action? Could the authors further argue and discuss this point?

11/ line 767: 'Experiments suggest that STN is involved in the cancellation of already initiated motor responses (Eagle and Robbins, 2003; Eagle et al., 2008)'. Another study in support of this statement is the work from Schmidt et al., 2013 DOI: 10.1038/nn.3456

12/ Please update the unpublished citation line 830 'Glajch et al., 2013' with the published one 'Glajch et al., 2016' DOI: 10.1523/JNEUROSCI.1720-15.2016

13/ The manuscript contain many typos, missing words, or punctuation errors. Here are the one I could spot upon reading:

-summary line 6: 'Recoding'

-Punctuation missing in Methods line 117: '...the axonal field of an FSN A one-dimensional...'

- Inappropriate repetition of the word 'respectively' line 205.

- Typo line 247: 'axonial'

-Line 264 'axonal three'

- Typo line 507 'subthalmo-pallidal network'

Fig 1 legend line 1305 'wheares'

-Line 592 the sentence starting 'Individually each....is another matter' is not clear. Please amend.

-Line 615 'Restoring the the..'

-Line 616 'decrese'..

-Line 625 'decease'..

-Line 635 'oscllations'

-Line 636 'dopmine'

-Line 614 'inscrease'

-Line 648 'in MNS D1' and 'wheares'.

-Line 664 'inhibititory'

- Line 698: 'implanted..'

-Unclear sentence in line 738: 'Thus it was with uncertainty and anticipation we ran..'

-Line 914: 'In particular it is explore to how..'

-Line 919 : 'predictiom'

- Contradictory sentence line 951: 'actually the conductance of STN-GPe TA synapses in our model is around four times weaker than STN-GPe TA connections'.
